# Comparative Risk of Hepatitis B Virus Reactivation in Patients Receiving Immune Checkpoint Inhibitors or Tyrosine Kinase Inhibitors for Liver Cancer

**DOI:** 10.1111/apt.70367

**Published:** 2025-09-05

**Authors:** Dorothy Cheuk‐Yan Yiu, Jimmy Che‐To Lai, Landon Long Chan, Grace Lai‐Hung Wong, Mandy Sze‐Man Lai, Vincent Wai‐Sun Wong, Yee‐Kit Tse, Henry Lik‐Yuen Chan, Stephen Lam Chan, Terry Cheuk‐Fung Yip

**Affiliations:** ^1^ Department of Medicine and Therapeutics The Chinese University of Hong Kong Hong Kong Hong Kong; ^2^ Medical Data Analytics Centre (MDAC) The Chinese University of Hong Kong Hong Kong Hong Kong; ^3^ Institute of Digestive Disease Hong Kong Hong Kong; ^4^ Faculty of Medicine, Li Ka Shing Institute of Health Sciences The Chinese University of Hong Kong Hong Kong Hong Kong; ^5^ State Key Laboratory of Translational Oncology, Department of Clinical Oncology The Chinese University of Hong Kong Hong Kong Hong Kong; ^6^ Department of Internal Medicine Union Hospital Hong Kong Hong Kong

**Keywords:** hepatitis B reactivation, hepatocellular carcinoma, immune checkpoint inhibitor, tyrosine kinase inhibitor

## Abstract

**Background:**

Current and past hepatitis B virus (HBV) infection remains the leading cause of liver cancer in endemic areas.

**Aim:**

To examine the risk of HBV reactivation (HBVr) in patients receiving immune checkpoint inhibitors (ICI) for liver cancer.

**Methods:**

Patients with current or past HBV infection receiving systemic treatments for liver cancer from March 2015 to March 2023 were identified using a territory‐wide electronic database in Hong Kong. The primary outcome was HBVr in ICI compared to tyrosine kinase inhibitor (TKI) use, defined according to the American Association for the Study of Liver Diseases criteria. The secondary outcome was HBVr in different types of ICI.

**Results:**

One thousand five hundred and ninty‐six patients with current or past HBV infection (222 first received ICI; 1374 first received TKI) were included. 205 patients (12.8%) had past HBV infection, and 93.2% of the cohort were on HBV antiviral prophylaxis at baseline. At a median of 10.7 months (IQR: 3.7–12.0), 25 (1.6%) patients had HBVr, among whom 5 were exposed to ICI. The 12‐month cumulative incidence (95% CI) of HBVr of the 1596 patients was 1.7% (1.1%–2.4%). The proportion of patients experiencing HBVr with and without antiviral prophylaxis was 1.4% and 3.7%, respectively. In multivariable analysis, ICI use was not associated with a higher risk of HBVr than TKI use, and the use of different ICI did not impact the risk of HBVr.

**Conclusion:**

With adequate antiviral prophylaxis, the absolute risk of HBVr is low in advanced HBV‐related liver cancer patients receiving ICI, regardless of current or past HBV infection.

## Graphical abstract

In a cohort of 1596 liver cancer patients with either current or prior HBV infection undergoing systemic therapy in Hong Kong, the risk of HBV reactivation during immune checkpoint inhibitor treatment was low when adequate antiviral prophylaxis was administered, irrespective of HBV infection status.

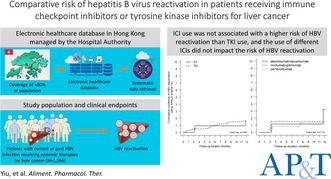

**Abbreviations**
AASLD: American Association for the Study of Liver Diseases
aCSHR: adjusted cause‐specific hazard ratio
ALT: alanine transaminase
anti‐HBs: hepatitis B surface antibody
APASL: Asian Pacific Association for the Study of the Liver
cccDNA: covalently closed circular DNA
CDARS: Clinical Data Analysis and Reporting System
CHB: chronic hepatitis B
CI: confidence interval
CTLA‐4: cytotoxic T‐lymphocyte‐associated protein 4
HBsAg: hepatitis B surface antigen
HBV: hepatitis B virus
HCC: hepatocellular carcinoma
HCV: hepatitis C virus
ICD‐9‐CM: International Classification of Diseases Ninth Revision Clinical Modification
IQR: inter‐quartile range
irAE: immune‐related adverse event
IS: immunosuppressants
LAT: local ablation therapy
NA: nucleos(t)ide analogues
PD‐(L)1: programmed cell death protein (ligand) 1
PD‐1: programmed cell death protein 1
sHR: subdistribution hazard ratio
TACE: transarterial chemoembolization
TKI: tyrosine kinase inhibitors

## 1 Introduction

Immune checkpoint inhibitors (ICI) have emerged as a promising treatment for patients with hepatocellular carcinoma (HCC). Since the IMBrave150 trials established atezolizumab–bevacizumab as the new standard of care in advanced HCC, ICI‐based regimens have become the mainstay of treatment for advanced HCC [1]. Despite the anti‐cancer efficacy of ICI, they are well known to induce various immune‐related adverse events (irAEs), most commonly affecting the skin, gastrointestinal tract, liver and endocrine glands [1]. They induce autoantibody production through effects on peripheral tolerance to autoantigens. In addition, they also promote autoimmunity and off‐tumour inflammation via stimulating pro‐inflammatory cytokine production in T‐cells [2].

Hepatitis B virus (HBV) reactivation is a known complication of anti‐tumour therapy such as conventional chemotherapy and targeted therapies. Patients with HCC are particularly vulnerable as they usually have impaired liver function. In addition, HBV infection is a major aetiology for HCC and accounts for 54% of HCC globally [3], implicating a large population at risk. In Hong Kong, the HBsAg seroprevalence in the general population is 6.2% in 2022 [4]. Chronic hepatitis B is the leading cause of HCC, accounting for 75%–80% of cases [5].

While HBV reactivation occurs due to immunosuppression from chemotherapy agents, the risk of ICI‐induced HBV reactivation remains unclear. It was hypothesised that ICI reinvigorates exhausted T‐cells to provide durable control of chronic infections including chronic hepatitis B (CHB) [6], yet paradoxically, HBV reactivation has also been reported in patients receiving ICI for both HCC and other tumours [6, 7, 8]. Programmed cell death protein 1 (PD‐1) and its ligand (PD‐L1) are important immunosuppressive mediators that maintain immune homeostasis and help prevent overwhelming hepatic damage. A possible explanation is that the blockade of the PD‐1/PD‐L1 axis led to the destruction of hepatocytes and further release of previously latent viruses into circulation. This suggests that ICI treatments may disrupt the balance of chronic HBV infection and lead to deterioration of liver function [6]. There are two main classes of ICI: anti‐PD‐1/PD‐L1 and anti‐cytotoxic T‐lymphocyte‐associated protein 4 (CTLA‐4) agents. While it was well recognised that anti‐CTLA‐4‐containing regimens increase the risk of irAEs [1], the difference between anti‐PD‐1 and anti‐CTLA‐4 use in HBV reactivation remains unclear.

HBV reactivation also acts as a negative prognostic indicator through deteriorating liver function and causing therapy interruption [9]. However, the exact incidence rate and risk factors of HBV reactivation in patients with advanced HCC receiving ICI remain uncertain. With the increasing popularity of different ICI‐based combination regimens (e.g., ICI plus tyrosine kinase inhibitor [TKI], double ICI regimens), the risk of HBV reactivation between different types of ICI should be explored. These important clinical questions should be addressed in a large cohort of patients in the regions with a high prevalence of HBV infection.

In this territory‐wide cohort study of patients with advanced liver cancer who had either current or past HBV infection, we aimed to investigate the risk of HBV reactivation among patients with liver cancer on ICI and compare the risk to those who received TKI, as well as to compare the risk of HBV reactivation among patients with liver cancer on different types of ICI.

## 2 Materials and Methods

### 2.1 Study Design and Data Source

We performed a retrospective territory‐wide cohort study using data from the Clinical Data Analysis and Reporting System (CDARS) under the management of the Hospital Authority, Hong Kong. CDARS facilitates the retrieval of clinical data captured from different operational systems for analysis and reporting and provides good‐quality information to support retrospective clinical and management decisions by integrating the clinical data residing in the Data Warehouse. It represents in‐patient and out‐patient data of approximately 80% of the 7.4‐million local population [10]. Patients are deidentified in CDARS to ensure confidentiality. Clinical data from CDARS have previously been used to conduct different territory‐wide studies on patients with liver cancer [11, 12, 13, 14]. The International Classification of Diseases, Ninth Revision, Clinical Modification (ICD‐9‐CM) and ICD‐10 coding systems were used in CDARS. Cross‐referencing with electronic medical records that include clinical, laboratory, imaging, and endoscopy results shows that ICD‐9‐CM codes in CDARS have a 99% accuracy rate in identifying medical diagnoses [15].

### 2.2 Subjects

We first identified all consecutive subjects ≥ 18 years old who received at least 1 dose of ICI or TKIs from March 2015 to March 2023 in Hong Kong. We excluded patients without liver cancer or exposure to HBV. Patients were followed from the date of the first prescription of ICI to the date of first HBV reactivation, death from any cause, last follow‐up date (3 May 2023), or 12 months of follow‐up, whichever came first. In the sensitivity analysis, patients were followed until 24 months of follow‐up. The study protocol was approved by the Joint Chinese University of Hong Kong–New Territories East Cluster Clinical Research Ethics Committee. Informed consent was waived given the retrospective nature of this study.

### 2.3 Data Collection

Data were retrieved from the CDARS in May 2023. Baseline date was defined as the date of the first prescription of ICI or TKI in the overall analysis, and the date of the first prescription of ICI in the analysis of different types of ICI. Demographic data including sex and date of birth were captured. At baseline, liver and renal biochemistries and haematological and virologic parameters (e.g., hepatitis B surface antigen [HBsAg], hepatitis B e antigen and its antibody, HBV DNA, antibody to hepatitis C virus [HCV], and HCV RNA) were collected (Table S1). Baseline liver biochemistries were defined as those results obtained immediately before the first dose of systemic treatment. Thereafter, serial liver biochemistries, HBV DNA, and HBsAg were collected until 3 May 2023. We also retrieved data on other relevant diagnoses, procedures, concomitant drugs, laboratory parameters, and exposure to nucleos(t)ide analogues (NAs) and (pegylated)‐interferon.

### 2.4 Use of Systemic Agents and Other Therapies

The following ICIs were used by patients: CTLA‐4 antibodies (ipilimumab); PD‐1 antibodies (nivolumab and pembrolizumab); programmed death‐ligand 1 (PD‐L1) inhibitors (atezolizumab). These agents were used as monotherapy or in combination as follows: atezolizumab–bevacizumab, nivolumab–ipilimumab, and pembrolizumab. The following TKI monotherapies were used by patients: sorafenib, lenvatinib, and carbozantinib. The procedures and use of medications before baseline were referred to record within 12 months before baseline.

### 2.5 HBV Infection and HBV Reactivation

Current HBV infection was defined with positive HBsAg and/or diagnosis codes. Past HBV infection was defined with negative HBsAg and positive hepatitis B core antibody. The primary outcome was HBV reactivation defined according to the American Association for the Study of Liver Diseases (AASLD) criteria. In patients with current HBV infection, HBV reactivation was defined as ≥ 1000 IU/mL, ≥ 10,000 IU/mL, or ≥ 100‐fold increase in HBV DNA with undetectable, unknown, and detectable baseline HBV DNA, respectively. In patients with past HBV infection, HBV reactivation is defined as the development of detectable HBV DNA and/or HBsAg [16]. The key secondary outcome was HBV reactivation based on the Asian Pacific Association for the Study of the Liver (APASL) guideline (i.e., current HBV infection: ≥ 100 IU/mL, ≥ 20,000 IU/mL, or ≥ 100‐fold increase with undetectable, unknown, and detectable baseline HBV DNA, respectively; past HBV infection: development of detectable HBV DNA and/or HBsAg) [17]. Other secondary outcomes included hepatitis flare, hepatic decompensation, and HBsAg seroclearance. Hepatitis flare was defined as alanine aminotransferase (ALT) > 5× upper limit of normal (ULN) if baseline ALT was normal; or > 5× baseline if baseline ALT was abnormal, based on Common Terminology Criteria for Adverse Events (CTCAE) v5.0. ULN was set at 40 U/L. Hepatitis flares considered HBV‐related were hepatitis flares accompanied by any of the following: a ≥ 1‐log increase in HBV DNA from baseline when HBV DNA was detectable at baseline; any newly detectable HBV DNA when it was undetectable at baseline; HBV DNA > 2000 IU/mL when baseline HBV DNA was unknown; or any detectable HBV DNA in individuals with past HBV infection. Hepatic decompensation was defined as occurrence of ascites, variceal bleeding, hepatic encephalopathy, spontaneous bacterial peritonitis, and/or hepatorenal syndrome defined by ICD‐9‐CM or ICD‐10 diagnosis codes or procedure codes (Table S2). HBsAg seroclearance was defined as undetectable serum HBsAg for at least once.

### 2.6 Antiviral Therapy

NA‐treated patients were defined as those prescribed and dispensed one or more NAs for CHB (i.e., lamivudine, adefovir dipivoxil, entecavir, telbivudine, tenofovir disoproxil fumarate, or tenofovir alafenamide) for any duration. Interferon exposure was defined by the dispensing record of interferon alpha‐2a/2b or peginterferon alpha‐2a/2b/lambda‐1a. These medications were identified by the Hospital Authority's internal drug codes (Table S3).

### 2.7 Statistical Analysis

Data were analysed using Statistical Product and Service Solutions version 25.0 (IBM, Armonk, NY) and R software (4.3.1; R Foundation for Statistical Computing, Vienna, Austria). Continuous variables were expressed in mean ± standard deviation or median (interquartile range [IQR]), as appropriate, whereas categorical variables were presented as number (percentage). Differences between groups were analysed by chi‐square or Fisher's exact tests for categorical parameters and Student's *t* test or Mann–Whitney test for continuous parameters, as appropriate. Cumulative incidence function with 95% confidence interval (CI) of primary and secondary endpoints was estimated with death as a competing risk and compared by Grey's test. Fine and Grey subdistribution hazard model was used to estimate the subdistribution hazard ratios (sHRs) of risk factors contributing to the development of HBV reactivation, with death as a competing risk. We used cause‐specific hazard regression to examine the association between the change of systemic therapies using a time‐dependent covariate and the development of HBV reactivation. The covariates examined in the regression model included patient demographics (age, sex, past or current HBV infection, use of HBV antiviral prophylaxis, steroid/immunosuppressant use before ICI), biochemical variables (baseline ALT, HBV DNA levels), and types of anti‐cancer treatment received (prior chemotherapy, prior transarterial chemoembolization (TACE), different ICI [pembrolizumab, atezolizumab plus bevacizumab, nivolumab plus ipilimumab]). A backward elimination method was used to select important factors in multivariable analysis. Multiple imputation by chained equations was used to account for missing data on covariates to create 20 complete data sets after the initial 10 iterations [18], assuming data were missing at random. The imputed baseline covariates (missing percentage) were ALT (0.1%) and HBV DNA (23.9%) at ICI. The covariates included in the imputation model were those examined in the regression model, HBV reactivation occurrence, and the Nelson–Aalen estimator of cumulative hazard at the time of HBV reactivation or censoring [19]. Imputed values were constrained within feasible ranges. Rubin's rules were applied to pool coefficient estimates and standard errors in each multiple imputation dataset for the overall estimates. All statistical tests were 2‐sided. Statistical significance was taken as *p* < 0.05.

## 3 Results

### 3.1 Demographic Characteristics

**FIGURE 1:**
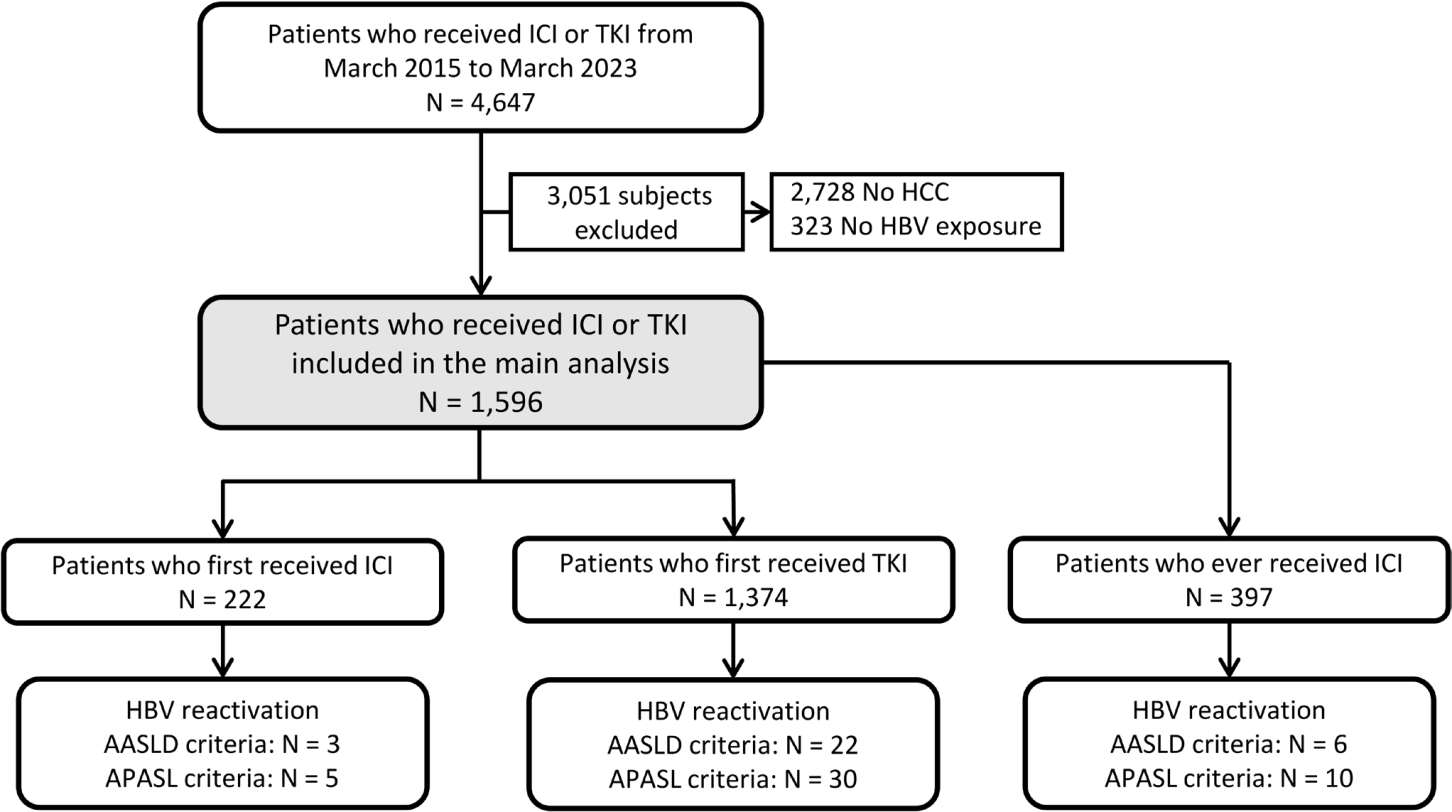
Selection of patients with current or past hepatitis B virus (HBV) infection and liver cancer who received immune checkpoint inhibitors (ICI) or tyrosine kinase inhibitors (TKIs). AASLD, American Association for the Study of Liver Diseases; APASL, Asian Pacific Association for the Study of the Liver.

We identified 4647 patients who had received ICI or TKI between March 2015 and March 2023; 2728 patients without liver cancer were excluded. Among 1919 patients with liver cancer, 1596 (83.2%) had current or past HBV infection, while another 323 patients without HBV exposure were excluded. Finally, 1596 patients with current or past HBV infection (222 received ICI, 1374 received TKI) were included and followed for a median of 10.7 months (IQR: 3.7–12.0) (Figure 1).

The cohort was male predominant (*n* = 1366, 85.6%), with a mean age of 63.3 ± 10.7 years; 511 (32.0%) had other cancers (Table S4); 397 (24.9%) had received steroids or other immunosuppressants within 12 months before receiving ICI or TKI, and the median duration of the use of steroids and other immunosuppressants was 5.0 (IQR: 1.1–9.9) months and 11.0 (IQR: 9.1–11.7) months, respectively. A total of 1391 (87.2%) and 205 patients (12.8%) had current and past HBV infection, respectively. The mean HBV DNA level was 3.8 ± 4.8 log_10_ IU/mL, with 93.2% of the cohort receiving HBV antiviral prophylaxis. Specifically, 1379 (99.1%) patients with current HBV infection and 108 (52.7%) with past HBV infection received HBV antiviral prophylaxis. 155 (9.7%), 84 (5.3%), and 541 (33.9%) patients had prior liver resection, local ablative therapy, and TACE before baseline, respectively. Baseline characteristics were similar between the two groups, except that more patients from the TKI group had prior TACE (Table 1).

**TABLE 1:** Clinical characteristics of patients with current or past hepatitis B virus (HBV) infection and hepatocellular carcinoma who received immune checkpoint inhibitors (ICI) or tyrosine kinase inhibitor (TKI).

Clinical characteristics	All *N* = 1596	ICI *N* = 222	TKI *N* = 1374	*p*
Age (years)	63.3 ± 10.7	62.7 ± 10.9	63.4 ± 10.7	0.360
Men, *n* (%)	1366 (85.6)	190 (85.6)	1176 (85.6)	0.999
Presence of other cancers, *n* (%)	511 (32.0)	61 (27.5)	450 (32.8)	0.118
HBV infection, *n* (%)				
Current HBV	1391 (87.2)	191 (86.0)	1200 (87.3)	0.591
Past HBV	205 (12.8)	31 (14.0)	174 (12.7)	
HBV antiviral prophylaxis, *n* (%)	1487 (93.2)	216 (97.3)	1271 (92.5)	0.009
HCV coinfection, (%)	61 (5.2)	6 (3.7)	55 (5.5)	0.341
Alanine aminotransferase (U/L)	38 (25–64)	40 (28–73)	38 (25–63)	0.059
Missing (%)	0.1	0.5	0	
HBV DNA level of patients with current HBV infection (log_10_ IU/mL)	4.2 ± 4.9	3.7 ± 4.4	4.2 ± 5.0	0.281
Missing (%)	22.0	28.8	20.9	
Use of liver cancer TKI at baseline, *n* (%)	1374 (86.1)	0 (0)	1374 (100)	< 0.001
Switch to liver cancer TKI in follow‐up, *n* (%)	71 (4.4)	71 (32.0)	0 (0)	< 0.001
Use of ICI at baseline, *n* (%)	222 (13.9)	222 (100)	0 (0)	< 0.001
Switch to ICI in follow‐up, *n* (%)	175 (11.0)	0 (0)	175 (12.7)	< 0.001
Use of other target therapies before baseline, *n* (%)	17 (1.1)	1 (0.5)	16 (1.2)	0.494
Start of other target therapies in follow‐up, *n* (%)	142 (8.9)	26 (11.7)	116 (8.4)	0.112
Use of other chemotherapy before baseline, *n* (%)	555 (34.8)	74 (33.3)	481 (35.0)	0.627
Start of other chemotherapy in follow‐up, *n* (%)	73 (4.6)	15 (6.8)	58 (4.2)	0.093
Liver resection before baseline, *n* (%)	155 (9.7)	21 (9.5)	134 (9.8)	0.891
LAT before baseline, *n* (%)	84 (5.3)	8 (3.6)	76 (5.5)	0.233
TACE before baseline, *n* (%)	541 (33.9)	62 (27.9)	479 (34.9)	0.043
Use of steroid/other IS before baseline, *n* (%)	397 (24.9)	48 (21.6)	349 (25.4)	0.227
Start of steroid/other IS in follow‐up, *n* (%)	315 (19.7)	41 (18.5)	274 (19.9)	0.609
Follow‐up duration (months)	10.7 (3.7–12.0)	8.4 (2.2–12.0)	11.0 (4.1–12.0)	0.004

Abbreviations: HBV, hepatitis B virus; HCV, hepatitis C virus; IS, immunosuppressants; LAT, local ablation therapy; TACE, transarterial chemoembolization; TKI, tyrosine kinase inhibitors.

### 3.2 HBV Reactivation

Using the AASLD criteria [16], 25 (1.6%) patients developed HBV reactivation, of which 8 and 17 patients had current and past HBV infection, respectively (Table 2); the 12‐month cumulative incidence (95% CI) of HBV reactivation was 1.7% (1.1%–2.4%). Table S5 shows the clinical characteristics of the 25 patients at the time of HBV reactivation. No patients stopped or changed to other ICI or TKI within 1 month after HBV reactivation. Two patients developed ascites after HBV reactivation. Using the AASLD criteria, the 12‐month cumulative incidence (95% CI) of HBV reactivation was 1.2% (0.5%–2.6%) and 1.9% (1.2%–2.9%) in patients with and without other cancers, respectively. The 12‐month cumulative incidence (95% CI) of HBV reactivation was 0.6% (0.3%–1.2%) in patients with current HBV infection, and 9.2% (5.5%–13.9%) in patients with past HBV infection, respectively. Among 17 patients with past HBV infection who developed HBV reactivation, 13 patients had received prior antiviral prophylaxis. All 8 patients with current HBV infection who developed HBV reactivation received antiviral therapy. 35 patients (2.2%) had HBV reactivation according to the definition of APASL guideline [17], including 18 current and 17 past HBV patients; the 12‐month cumulative incidence (95% CI) of HBV reactivation was 2.4% (1.7%–3.2%). Compared with patients without HBV reactivation, patients who developed HBV reactivation were more likely to have past HBV infection and had lower HBV DNA levels at baseline (Table 2, Table S6). Using the APASL criteria, the 12‐month cumulative incidence (95% CI) of HBV reactivation was 0.6% (0.1%–2.1%) in patients with current HBV infection, and 11.9% (3.5%–25.9%) in patients with past HBV infection, respectively.

**TABLE 2:** Clinical characteristics of patients with current or past hepatitis B virus (HBV) infection and hepatocellular carcinoma who received immune checkpoint inhibitors (ICI) or tyrosine kinase inhibitor (TKI) and did or did not develop HBV reactivation based on the American Association for the Study of Liver Diseases criteria.

Clinical characteristics	Without HBV reactivation *N* = 1571	With HBV reactivation *N* = 25	*p*
Age (years)	63.3 ± 10.8	62.1 ± 7.7	0.577
Men, *n* (%)	1343 (85.5)	23 (92.0)	0.565
Presence of other cancers, *n* (%)	505 (32.1)	6 (24.0)	0.387
HBV infection, *n* (%)			
Current HBV	1383 (88.0)	8 (32.0)	< 0.001
Past HBV	188 (12.0)	17 (68.0)	
HBV antiviral prophylaxis, *n* (%)	1466 (93.3)	21 (84.0)	0.086
HCV coinfection (%)	61 (5.3)	0 (0)	0.622
Alanine aminotransferase (U/L)	38 (25–64)	48 (31–93)	0.222
Missing (%)	0.1	0	
HBV DNA level of patients with current HBV infection (log_10_ IU/mL)	4.2 ± 4.9	1.9 ± 2.2	0.358
Missing (%)	21.8	50.0	
Use of liver cancer TKI at baseline, *n* (%)	1352 (86.1)	22 (88.0)	> 0.999
Switch to liver cancer TKI in follow‐up, *n* (%)	71 (4.5)	0 (0)	0.624
Use of ICI at baseline, *n* (%)	219 (13.9)	3 (12.0)	> 0.999
Switch to ICI in follow‐up, *n* (%)	173 (11.0)	2 (8.0)	> 0.999
Use of other target therapies before baseline, *n* (%)	17 (1.1)	0 (0)	> 0.999
Start of other target therapies in follow‐up, *n* (%)	142 (9.0)	0 (0)	0.160
Use of other chemotherapy before baseline, *n* (%)	549 (34.9)	6 (24.0)	0.254
Start of other chemotherapy in follow‐up, *n* (%)	72 (4.6)	1 (4.0)	> 0.999
Liver resection before baseline, *n* (%)	152 (9.7)	3 (12.0)	0.728
LAT before baseline, *n* (%)	83 (5.3)	1 (4.0)	> 0.999
TACE before baseline, *n* (%)	535 (34.1)	6 (24.0)	0.292
Use of steroid/IS before baseline, *n* (%)	390 (24.8)	7 (28.0)	0.716
Start of steroid/IS in follow‐up, *n* (%)	312 (19.9)	3 (12.0)	0.450
Follow‐up duration (months)	10.9 (3.8–12.0)	5.1 (1.8–8.2)	< 0.001

Abbreviations: HBV, hepatitis B virus; HCV, hepatitis C virus; IS, immunosuppressants; LAT, local ablation therapy; TACE, transarterial chemoembolization; TKI, tyrosine kinase inhibitors.

Among 1487 patients who received antiviral prophylaxis, 21 (1.4%) patients developed HBV reactivation; while 4/109 (3.7%) patients who did not receive HBV prophylaxis developed HBV reactivation. Among the 21 patients, 8 and 13 patients had current and past HBV infection, respectively; 19 patients were receiving TKI (16 on lenvatinib, 3 on sorafenib) when HBV reactivation occurred, with the remaining 2 receiving ICI‐TKI combination regimen (atezolizumab–bevacizumab). The median duration of antiviral prophylaxis before the development of HBV reactivation is 449 days (IQR: 169–771 days). Four out of eight patients with current HBV infection had baseline HBV DNA measurement, two were undetectable while two had levels of 37 and 52 IU/mL, respectively.

Among the 8 and 13 patients with current and past HBV infection who received antiviral prophylaxis, there was a median rise of 2.4 folds (IQR: 1.2–8.5) and 2.2 folds (IQR: 1.6–4.7) in ALT levels, respectively. The median maximum ALT level at HBV reactivation was 219 (range: 65–5706) and 134 (range: 28–827) U/L, respectively. The median maximum HBV DNA level at HBV reactivation was 4.4 (range: 3.1–6.3) and 1.9 (range: 1.3–3.9) log_10_ IU/mL, respectively. Of the four patients who did not receive antiviral prophylaxis, all had past HBV infection. Their ALT levels rose by 2.1, 2.5, 3.2 and 12.5 times to a maximum level of 34, 280, 289 and 388 U/L, respectively during HBV reactivation. Two had quantifiable HBV DNA levels of 1.59 and 1.71 log_10_ IU/mL. HBV reactivation occurred in 4/387 (1.0%) patients receiving ICI with antiviral prophylaxis and 2/10 (20.0%) patients receiving ICI without antiviral prophylaxis. Four ICI patients with past HBV infection developed HBV reactivation, in which two of them received antiviral prophylaxis.

**FIGURE 2:**
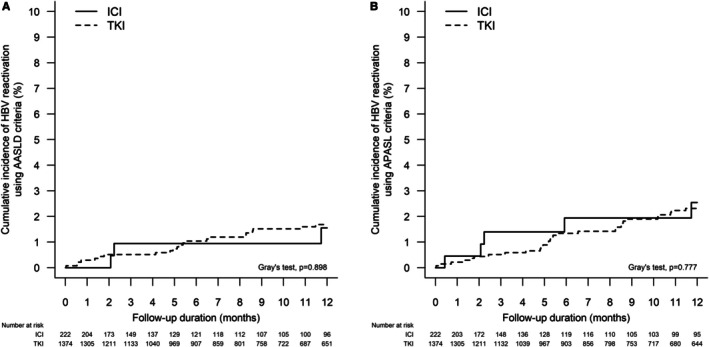
Cumulative incidence of hepatitis B virus (HBV) reactivation A. using American Association for the Study of Liver Diseases (AASLD) criteria; and B. using APASL criteria in patients with current or past HBV infection and liver cancer who received immune checkpoint inhibitors (ICI) or tyrosine kinase inhibitors (TKI).

The 12‐month cumulative incidence (95% CI) of HBV reactivation using AASLD criteria in patients who received ICI and TKI is 1.6% (0.4%–4.2%) and 1.7% (1.1%–2.5%), respectively (Grey's test, *p* = 0.898) (Figure 2A). The 12‐month cumulative incidence (95% CI) of HBV reactivation using APASL criteria in patients who received ICI and TKI is 2.5% (0.9%–5.6%) and 2.3% (1.6%–3.2%), respectively (Grey's test, *p* = 0.777) (Figure 2B). In multivariable analysis, the use of ICI was not associated with a higher risk of HBV reactivation than the use of TKI (subdistribution hazard ratio [sHR] 1.03, 95% CI: 0.30–3.54, *p* = 0.962), after adjusting for HBV antiviral prophylaxis; a similar result was observed when using the APASL criteria for HBV reactivation (sHR 0.86 [95% CI: 0.33–2.25], *p* = 0.762) (Table S7).

We investigated the impact of changing from ICI to TKI and vice versa on the risk of HBV reactivation using time‐dependent cause‐specific hazard regression. In multivariable analyses, compared with the use of TKI alone, patients who used ICI (adjusted cause‐specific hazard ratio [aCSHR] 1.31, 95% CI: 0.39–4.43, *p* = 0.666) or switched between ICI and TKI (aCSHR 1.65, 95% CI: 0.38–7.27, *p* = 0.507) were not associated with a higher risk of HBV reactivation. Patients who switched from ICI to TKI or vice versa had a higher risk of death (aCSHR 2.13, 95% CI: 1.63–2.77, *p* < 0.001). Other risk factors of death included the use of steroids or immunosuppressants (aCSHR 1.59 [95% CI: 1.36–1.87], *p* < 0.001); baseline HBV DNA (aCSHR 1.07 [1.05–1.08]); and baseline ALT levels (aCSHR 1.003 [1.002–1.004], *p* < 0.001) (Table S8).

### 3.3 HBV Reactivation in Different Types of ICI

Including 175 patients who switched from TKI to ICI in subsequent follow‐up, we identified 397 patients who had ever received ICI (Figure 1); 236 (59.9%) patients received pembrolizumab, 96 (24.2%) received atezolizumab–bevacizumab and 65 received nivolumab–ipilimumab (16.4%). The nivolumab–ipilimumab group had the highest baseline ALT, followed by atezolizumab–bevacizumab and pembrolizumab patients. More patients from the pembrolizumab group had prior liver cancer targeted therapy, while a larger proportion of atezolizumab–bevacizumab and nivolumab–ipilimumab groups had subsequent liver cancer targeted therapy (Table 3).

**TABLE 3:** Clinical characteristics of patients with current or past hepatitis B virus (HBV) infection and hepatocellular carcinoma who received atezolizumab plus bevacizumab, nivolumab plus ipilimumab, or pembrolizumab.

Clinical characteristics	All *N* = 397	Atezolizumab + bevacizumab *N* = 96	Nivolumab + ipilimumab *N* = 65	Pembrolizumab *N* = 236	*p*
Age (years)	62.1 ± 11.2	63.4 ± 9.6	60.8 ± 13.3	61.9 ± 11.2	0.319
Men, *n* (%)	339 (85.4)	81 (84.4)	57 (87.7)	201 (85.2)	0.833
Presence of other cancers, *n* (%)	130 (32.7)	23 (24.0)	21 (32.3)	86 (36.4)	0.089
HBV infection, *n* (%)					
Current HBV	355 (89.4)	80 (83.3)	61 (93.8)	214 (90.7)	0.064
Past HBV	42 (10.6)	16 (16.7)	4 (6.2)	22 (9.3)	
HBV antiviral prophylaxis, *n* (%)	387 (97.5)	94 (97.9)	64 (98.5)	229 (97.0)	> 0.999
HCV coinfection, *n* (%)	10 (3.4)	2 (2.8)	2 (4.3)	6 (3.4)	0.907
Alanine aminotransferase (U/L)	41 (26–69)	42 (27–59)	50 (35–84)	38 (25–70)	0.010
Missing (%)	0.3	0	0	0.4	
HBV DNA level (log_10_ IU/mL)	3.2 ± 4.2	2.6 ± 3.6	4.2 ± 5.0	3.3 ± 4.3	0.135
Missing (%)	25.2	16.7	29.2	27.5	
HBV DNA level of patients with current HBV infection (log_10_ IU/mL)	3.6 ± 4.3	2.9 ± 3.8	4.5 ± 5.1	3.6 ± 4.3	0.161
Missing (%)	24.8	15	29.5	27.1	
Switch of ICI in follow‐up, *n* (%)	141 (35.5)	37 (38.5)	24 (36.9)	80 (33.9)	0.701
Use of liver cancer TKI before ICI, *n* (%)	164 (41.3)	15 (15.6)	19 (29.2)	130 (55.1)	< 0.001
Start of liver cancer TKI in follow‐up, *n* (%)	75 (18.9)	28 (29.2)	14 (21.5)	33 (14.0)	0.005
Use of other target therapies before ICI, *n* (%)	12 (3.0)	1 (1.0)	2 (3.1)	9 (3.8)	0.490
Start of other target therapies in follow‐up, *n* (%)	54 (13.6)	11 (11.5)	12 (18.5)	31 (13.1)	0.422
Use of other chemotherapy before ICI, *n* (%)	113 (28.5)	31 (32.3)	14 (21.5)	68 (28.8)	0.327
Start of other chemotherapy in follow‐up, *n* (%)	22 (5.5)	4 (4.2)	3 (4.6)	15 (6.4)	0.783
Liver resection before ICI, *n* (%)	34 (8.6)	10 (10.4)	6 (9.2)	18 (7.6)	0.697
LAT before ICI, *n* (%)	15 (3.8)	3 (3.1)	2 (3.1)	10 (4.2)	0.934
TACE before ICI, *n* (%)	102 (25.7)	32 (33.3)	15 (23.1)	55 (23.3)	0.144
Use of steroid/IS before ICI, *n* (%)	94 (23.7)	22 (22.9)	6 (9.2)	66 (28.0)	0.007
Start of steroid/IS in follow‐up, *n* (%)	90 (22.7)	14 (14.6)	19 (29.2)	57 (24.2)	0.065
Follow‐up duration (months)	6.8 (2.2–12.0)	7.4 (3.0–12.0)	3.6 (1.7–12.0)	7.1 (2.3–12.0)	0.140

Abbreviations: HBV, hepatitis B virus; HCV, hepatitis C virus; ICI, immune checkpoint inhibitors; IS, immunosuppressants; LAT, local ablation therapy; TACE, transarterial chemoembolization; TKI, tyrosine kinase inhibitors.

**FIGURE 3:**
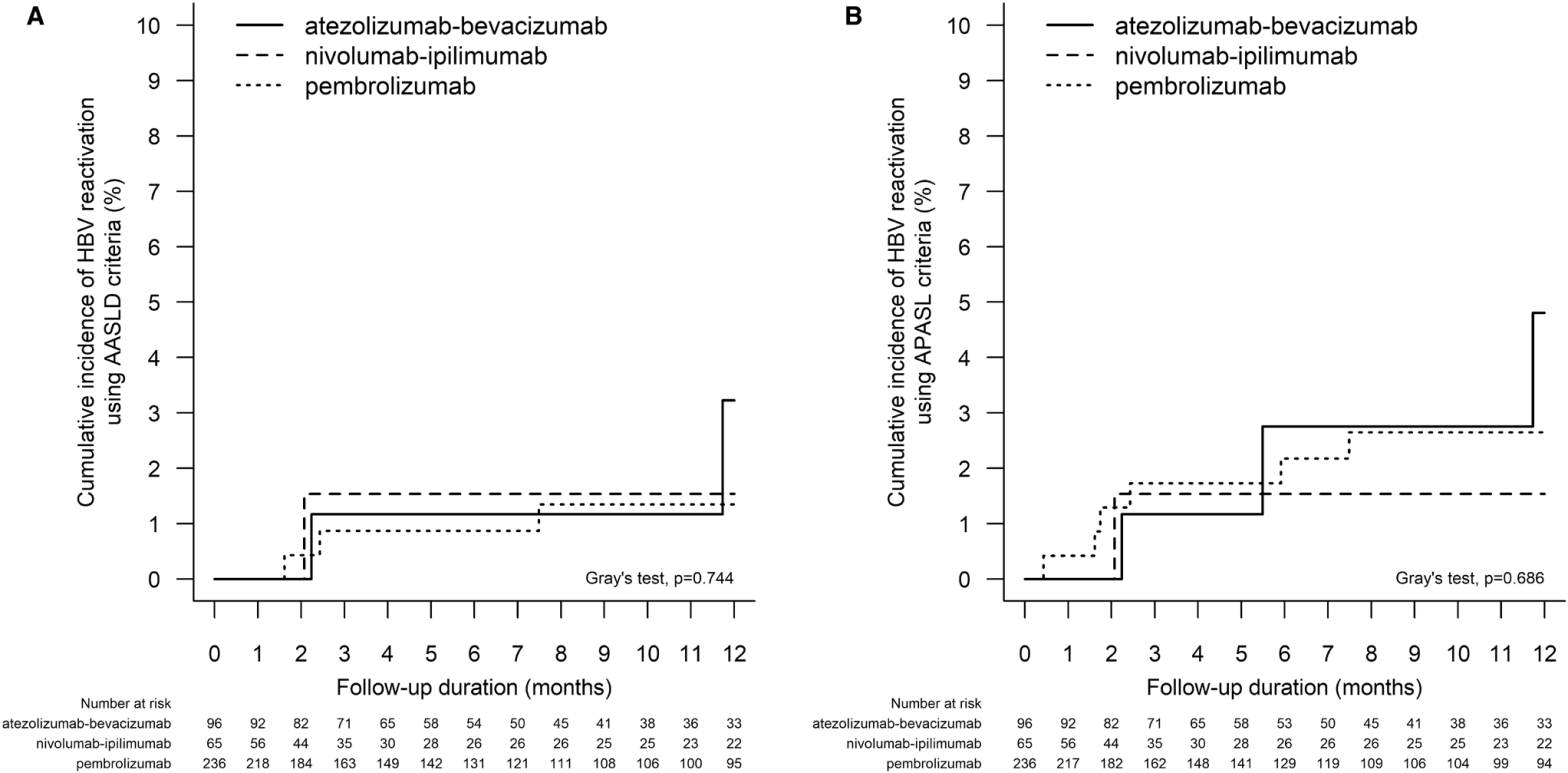
Cumulative incidence of hepatitis B virus (HBV) reactivation A. using American Association for the Study of Liver Diseases (AASLD) criteria; and B. using APASL criteria in patients with current or past HBV infection and liver cancer who received atezolizumab–bevacizumab, nivolumab–ipilimumab, and pembrolizumab.

At a median follow‐up of 6.8 (IQR: 2.2–12.0) months, 6 (1.5%) and 10 (2.5%) patients developed HBV reactivation according to AASLD and APASL criteria, respectively (Tables S9 and S10). The 12‐month cumulative incidence (95% CI) of HBV reactivation using AASLD criteria in patients who received atezolizumab–bevacizumab, nivolumab–ipilimumab, and pembrolizumab is 3.2% (0.5%–10.4%), 1.5% (0.1%–7.4%), and 1.3% (0.4%–3.6%), respectively (Grey's test, *p* = 0.744) (Figure 3A). The 12‐month cumulative incidence (95% CI) of HBV reactivation using APASL criteria in patients who received atezolizumab–bevacizumab, nivolumab–ipilimumab, and pembrolizumab is 4.8% (1.2%–12.5%), 1.5% (0.1%–7.4%), and 2.6% (1.1%–5.4%), respectively (Grey's test, *p* = 0.686) (Figure 3B).

In multivariable analysis, the use of different types of ICI was not associated with a significant difference in the risk of developing HBV reactivation (atezolizumab–bevacizumab vs. pembrolizumab: aSHR 0.64 [95% CI: 0.10–4.04], *p* = 0.636; atezolizumab–bevacizumab vs. pembrolizumab: aSHR 0.47 [95% CI: 0.07–3.13], *p* = 0.432). We identified the use of HBV antiviral prophylaxis (aSHR 0.03, 95% CI: 0.01–0.15, *p* < 0.001) as a major protective factor against HBV reactivation. TACE before ICI significantly increased the risk of HBV reactivation (aSHR 8.54, 95% CI: 1.22–59.60, *p* = 0.031) (Table 4). A similar finding was shown when using the APASL criteria to define HBV reactivation (Table 4). In multivariable analysis, the switch in IO treatments did not correlate with a higher incidence of HBV reactivation but a higher mortality (Table S11).

**TABLE 4:** Univariate and multivariable analysis with Fine‐Grey subdistribution hazard regression after multiple imputation on factors associated with the development of HBV reactivation in patients with current or past infection of hepatitis B and hepatocellular carcinoma who received atezolizumab plus bevacizumab, nivolumab plus ipilimumab, or pembrolizumab.

Parameters	Univariate analysis		Multivariable analysis	
	sHR (95% CI)	*p*	aSHR (95% CI)	*p*
HBV reactivation using AASLD criteria (reactivation rate = 6/397 [1.5%])				
Use of different ICI				
Pembrolizumab	Reference			
Atezolizumab + bevacizumab	0.61 (0.05–7.09)	0.692	0.64 (0.10–4.04)	0.636
Nivolumab + ipilimumab	0.52 (0.08–3.18)	0.477	0.47 (0.07–3.13)	0.432
Past vs. current HBV infection	19.70 (3.71–104.68)	< 0.001		
HBV antiviral prophylaxis	0.14 (0.03–0.80)	0.027	0.03 (0.01–0.15)	< 0.001
Age	1.04 (0.97–1.12)	0.270		
Men^a^	N.A.	—		
Presence of other cancers	0.98 (0.18–5.26)	0.979		
ALT at ICI (U/L)	1.00 (1.00–1.01)	0.442		
HBV DNA at ICI (log_10_ IU/mL)	0.98 (0.87–1.10)	0.708		
Other chemotherapy before ICI	5.09 (0.94–27.70)	0.060		
TACE before ICI	6.15 (1.13–33.39)	0.036	8.54 (1.22–59.60)	0.031
Steroid/immunosuppressant use before ICI	3.05 (0.64–14.50)	0.161		
HBV reactivation using APASL criteria (reactivation rate = 10/397 [2.5%])				
Use of different ICI				
Pembrolizumab	Reference			
Atezolizumab + bevacizumab	0.41 (0.04–4.05)	0.443	0.48 (0.04–5.18)	0.545
Nivolumab + ipilimumab	0.69 (0.17–2.84)	0.609	0.69 (0.17–2.87)	0.608
Past vs. current HBV infection	6.51 (1.87–22.65)	0.003		
HBV antiviral prophylaxis	0.10 (0.02–0.46)	0.003	0.10 (0.02–0.57)	0.010
Age	1.02 (0.97–1.06)	0.504		
Men^a^	N.A.	—		
Presence of other cancers	0.49 (0.10–2.26)	0.358		
ALT at ICI (U/L)	1.00 (0.99–1.01)	0.688		
HBV DNA at ICI (log_10_ IU/mL)	0.89 (0.76–1.04)	0.130		
Other chemotherapy before ICI	2.55 (0.74–8.74)	0.137		
TACE before ICI	3.06 (0.89–10.54)	0.076		
Steroid/immunosuppressant use before ICI	1.31 (0.35–4.88)	0.692		

Abbreviations: AASLD, American Association for the Study of Liver Diseases; ALT, alanine transaminase; APASL, Asian Pacific Association for the Study of the Liver; aSHR, adjusted subdistribution hazard ratio; HBV, hepatitis B virus; ICI, immune checkpoint inhibitors; IS, immunosuppressants; TACE, transarterial chemoembolization; TKI, tyrosine kinase inhibitors.

^a^ All HBV reactivations occurred in men.

### 3.4 Hepatitis Flare

Among 1596 patients, 249 (15.6%) developed hepatitis flare within 12 months. Among the 249 patients who had hepatitis flare, 55 (22.0%) were considered HBV‐related, and 32 (12.9%) were prescribed steroids after the hepatitis flare; 3 (1.2%) stopped or changed to other ICI or TKI within 1 month after hepatitis flare. Among 222 patients who first received ICI and 1374 patients who first received TKI, 26 (11.7%) and 223 (16.2%) developed hepatitis flare within 12 months, respectively (Figure S1A; Grey's test, *p* = 0.234). The 12‐month cumulative incidence (95% CI) of hepatitis flare was 16.7% (14.7%–18.7%) in patients with current HBV infection and 15.0% (10.3%–20.6%) in patients with past HBV infection. Among 96, 65, and 236 patients who used atezolizumab/bevacizumab, nivolumab/ipilimumab, and pembrolizumab, 10 (10.4%), 10 (15.4%), and 37 (15.7%) developed hepatitis flare in 12 months (Figure S1B; Grey's test, *p* = 0.730).

### 3.5 Hepatic Decompensation

Among 1529 patients without hepatic decompensation at baseline, 419 (27.4%) developed hepatic decompensation within 12 months. Among 211 patients who first received ICI and 1318 patients who first received TKI, 52 (24.6%) and 367 (27.8%) developed hepatic decompensation within 12 months, respectively (Figure S2A; Grey's test, *p* = 0.951). Among 95, 55, and 219 patients without hepatic decompensation who used atezolizumab/bevacizumab, nivolumab/ipilimumab, and pembrolizumab, 11 (11.6%), 17 (30.9%), and 59 (26.9%) developed hepatic decompensation in 12 months (Figure S2B; Grey's test, *p* = 0.072).

### 3.6 HBsAg Seroclearance

Among 80, 61, and 214 patients with current HBV infection who received atezolizumab/bevacizumab, nivolumab/ipilimumab, and pembrolizumab, 3 (3.8%), 0 (0%), and 2 (0.9%) achieved HBsAg seroclearance, respectively.

## 4 Discussion

This territory‐wide cohort study investigated the incidence and risk factors of HBV reactivation in patients with advanced liver cancer in Hong Kong who received different types of systemic treatment including ICI and TKI. We demonstrated that the overall risk of HBV reactivation is low and is not affected by the choice of TKI or different types of ICI. However, patients with past HBV and prior TACE before ICI may be predisposed to the development of HBV reactivation. HBV antiviral prophylaxis remained important in preventing HBV reactivation.

Under good overall coverage of HBV antiviral prophylaxis, the absolute risk of HBV reactivation was low. In a meta‐analysis of 366 patients with HCC who received systemic therapy, the overall pooled HBV reactivation rates were 9% in 66 patients who did not receive HBV prophylaxis and 3% in 179 who did [20]. In another meta‐analysis on HBV reactivation during chemotherapy in patients with solid tumours, patients without antiviral protection had a median reactivation risk of 25% (range: 4%–68%) with chronic HBV infection, and 3% (range: 0.3%–9%) with past HBV infection. Antiviral prophylaxis notably decreased reactivation risk, thus lowering the chances of HBV‐related hepatitis flare and interruptions in chemotherapy [21]. This is consistent with our findings, in which 1.4% and 3.7% of patients with and without antiviral prophylaxis developed HBV reactivation. 93.2% of our cohort had received HBV prophylaxis, translating into a HBV reactivation incidence of 1.6%. The risk of HBV reactivation in patients with current HBV infection after ICI and TKI is moderate and high under the current American Gastroenterological Association (AGA) guideline, respectively [22]. Thus, HBV prophylaxis should be routinely offered to minimise the occurrences of reactivation events.

An important finding of this study is that the risk of HBV reactivation in ICI was comparable to that of TKI, and there was no difference in the risk of HBV reactivation between those who changed therapies from ICI to TKI and vice versa and those who received TKI only. While ICI could restore the function of exhausted virus‐specific CD8 T cells by PD‐1/PD‐L1 blockade, boosting immune control on HCC growth, it could also disrupt the immune homeostasis in the liver, leading to an increased immune response to hepatocytes and subsequent liver damage. It was also proposed that PD‐1 and CTLA‐4 blockade reduce regulatory T cells and impair protective immune responses, indirectly promoting HBV survival and expansion [6, 8]. With the opposite mechanistic pathways of ICIs on HBV reactivation and the coverage of HBV prophylaxis, the risk of HBV reactivation in ICI remains similar to that of TKI. Our findings were also in line with a recent study which estimated the risk of HBV reactivation in patients on ICI, which reported that HBV reactivation occurred at a rate of 0.5% (2/409) and 2.9% (3/102) in HBsAg positive patients with and without HCC, respectively [23]. In the subgroup analysis of patients who started with ICI, the risk of HBV reactivation among different types of ICI was similar. This suggests that double‐ICI combination regimens do not increase the risk of HBV reactivation, similar to that of irAEs. However, patients who changed systemic therapies had an increased risk of mortality, no matter whether it was a switch to a different class of anti‐cancer drugs (i.e., TKI to ICI or vice versa) or changing the type of ICI. This reflected a more aggressive or extensive HCC disease, as the most common indication for changing therapy is disease progression.

In our cohort, two‐thirds of patients who had HBV reactivation based on the AASLD criteria had past HBV infection. The incidence of HBV reactivation in these patients is higher in comparison with patients with current HBV infection. Our findings demonstrated that HBV reactivation remains possible in individuals who have past HBV infection when exposed to anti‐cancer treatments. This susceptibility is linked to the immunological processes that trigger HBV reactivation. Following HBV infection, liver cells retain covalently closed circular DNA (cccDNA) as a transcription template of HBV, even after HBsAg seroclearance, and cccDNA can lead to reactivation under immunosuppression. Consequently, individuals presumed to have past HBV infection, with negative HBsAg, detectable hepatitis B surface antibodies (anti‐HBs) and undetectable HBV DNA in their blood, are still at risk of reactivation. In addition, there could be false‐negative HBsAg test results due to mutant HBsAg or low HBsAg titres that may not be detected by common commercial assay of HBsAg [24].

Within patients with past HBV infection, the risk of HBV reactivation could be further stratified with anti‐HBs. Studies have suggested that those with detectable anti‐HBs show a relatively lower risk of HBV reactivation. In a prospective study evaluating the risk of HBV reactivation in past HBV patients undergoing chemotherapy for lymphoma, HBV reactivation occurred in 9/116 anti‐HBs positive patients (8%) and 8/35 (23%) anti‐HBs negative patients [25]. A meta‐analysis by Paul et al. [21] reported that the presence of anti‐HBs reduced the reactivation risk with a pooled odds ratio of 0.21 in the absence of HBV prophylactic treatment. This emphasises the necessity of screening all patients before commencing systemic treatments not just for HBsAg but also for anti‐HBs and hepatitis B core antibodies to assess their reactivation risk.

In this study, HBV prophylaxis was only used in 53% of patients with past HBV infection. In 17 patients with past HBV infection who developed HBV reactivation, 4 (23.5%) patients had not received antiviral treatment; while only two out of four ICI users with past HBV infection and HBV reactivation had antiviral prophylaxis, revealing a concerning gap in antiviral coverage in these patients. Although the AGA guidelines classify patients with resolved hepatitis B infection undergoing ICI therapy as low risk for HBV reactivation, our study observed a notable incidence of reactivation among this group [22]. The 12‐month cumulative incidence of HBV reactivation was 9.2% among patients with past HBV infection, which can be considered a moderate risk according to the AGA guideline. HBV antiviral prophylaxis over monitoring alone may be considered in these patients. On the choice of antiviral agents, both entecavir and tenofovir have shown good efficacy in suppressing HBV replication and preventing HBV reactivation in patients receiving immunosuppressive or cytotoxic agents, but data on ICIs remain lacking [6, 8]. However, the limitations of available antiviral agents in targeting cccDNA should be noted, as 84% of patients who developed HBV reactivation had been given HBV prophylaxis. HBV establishes a pool of cccDNA in the nucleus of infected hepatocytes during infection that acts as a persistent reservoir responsible for HBV reactivation even among patients with past HBV infection [26]. Although the risk of HBV reactivation could be greatly reduced through antiviral treatment, it could not be eliminated.

We identified TACE before ICI as a risk factor for HBV reactivation. TACE has been well reported as a risk factor for reactivation in HCC [9, 26, 27]. TACE induces immunosuppression and damages immune cells in the liver, allowing enhanced HBV replication. When the cytotoxic therapies were withdrawn, immune function was restored and the HBV‐infected hepatocytes were destroyed [27]. In the scenario of ICI agents, immune function is further enhanced through the reactivation of exhausted T cells, thus the increased destruction of HBV‐infected hepatocytes predisposes to HBV reactivation. The findings are also supported by a retrospective cohort study examining the risk of HBV reactivation in patients undergoing TACE combined with TKI‐ICI regimens. Within the entire cohort, 12/119 patients (10.1%) had HBV reactivation. Among those who had received antiviral prophylaxis, HBV reactivation occurred in 4/95 (4.2%) patients [26].

Our study possesses several strengths, including substantial sample sizes in both the TKI and ICI cohorts to investigate the incidence. Additionally, we collected comprehensive drug and laboratory data at various time points. Real‐world cohorts could better reflect and relate to everyday clinical practice, as they provide a broader range of patients compared to randomised controlled trials that often exclude individuals with multiple comorbidities.

The strength of our study includes the large cohort size of patients with liver cancer who received systemic therapies. The validated diagnosis and procedure coding system, comprehensive laboratory data and drug information facilitated the analysis of the impact of systemic therapies, demographics, laboratory parameters, and concomitant medications on HBV reactivation. Information derived from real‐world cohorts encompasses a broader range of patients compared to those involved in randomised trials, where those with multiple comorbidities are frequently not included. Therefore, findings from real‐world data are more directly relevant to daily clinical practice. Nonetheless, our study has a few limitations. First, limited by its retrospective nature, we have missing data, particularly for serum anti‐HBs, anti‐HBc, follow‐up HBsAg, and HBV DNA level, because they were not performed according to a predefined research protocol. Residual confounding may exist. The information on liver cancer staging was not available, while it is expected that most of the patients had BCLC stage C or above due to the need for systemic therapies. The information on patient adherence to HBV antiviral prophylaxis was not available. A meta‐analysis suggested that the overall adherence to antiviral treatment was around 75% in patients with CHB [28]. We acknowledge that some patients who developed HBV reactivation despite HBV antiviral prophylaxis may have poor drug adherence. Some patients with negative HBsAg may have undiagnosed past HBV infection and thus were not included in this study. This was partly counterweighed by our large cohort size. We also employed multiple imputation to avoid selection bias due to missing data. Second, the number of patients who were not on HBV prophylaxis is small; hence, further subgroup analysis on the risk of HBV reactivation based on prophylactic antiviral therapy use could not be performed. However, we believe that HBV prophylaxis remains necessary due to the non‐negligible risk of HBV reactivation despite a good coverage of prophylaxis. Moreover, the high coverage of HBV antiviral prophylaxis and the relatively short median follow‐up duration of 11 months resulted in a low incidence of HBV reactivation. Further subgroup analyses on the clinical features of patients with HBV reactivation could not be conducted. Third, we were not able to accurately identify the indications for the use of steroids and other immunosuppressants. Fourth, we were not able to completely differentiate between immune‐related hepatitis and HBV‐related hepatitis flares. Among patients with hepatitis flare within 12 months of ICI or TKI, 22.0% were considered HBV‐related, 12.9% were prescribed steroids after hepatitis flare, and 1.2% stopped or changed to other ICI or TKI. Fifth, 32% of the patients with liver cancer had other cancers. We acknowledge that some patients classified as having HCC may have been misclassified and instead had liver metastases originating from other primary cancers. Also, some patients who were initially coded as malignant neoplasm of other and unspecified sites may later be confirmed as HCC. Last, unmeasured factors might have predisposed to confounding; hence we implemented rigorous exclusion criteria and conducted multivariable analysis to minimise bias.

In summary, HBV‐related liver cancer patients receiving ICI may be at risk of HBV reactivation, especially those with a history of TACE for liver cancer and without NA prophylaxis. The absolute risk of HBV reactivation was low in advanced liver cancer patients receiving TKI and ICI, under good overall coverage of HBV prophylaxis. Clinicians should closely monitor these patients and consider provision of NA prophylaxis to both patients with current and past HBV infection prior to treatment, to mitigate the potential risk of HBV reactivation. Further prospective studies should be conducted to elucidate the risk of HBV reactivation in past HBV infection.

## Author Contributions

**Dorothy Cheuk‐Yan Yiu:** writing – review and editing, writing – original draft, formal analysis, methodology. **Jimmy Che‐To Lai:** writing – review and editing, methodology, supervision. **Landon Long Chan:** methodology, writing – review and editing. **Grace Lai‐Hung Wong:** methodology, writing – review and editing, supervision, data curation. **Mandy Sze‐Man Lai:** methodology, writing – review and editing, visualization, formal analysis. **Vincent Wai‐Sun Wong:** methodology, writing – review and editing, supervision. **Yee‐Kit Tse:** methodology, writing – review and editing. **Henry Lik‐Yuen Chan:** methodology, writing – review and editing, supervision. **Stephen Lam Chan:** methodology, writing – review and editing, supervision. **Terry Cheuk‐Fung Yip:** writing – original draft, writing – review and editing, methodology, conceptualization, visualization, supervision, formal analysis.

## Conflicts of Interest

Grace Wong has served as an advisory committee member for AstraZeneca, Gilead Sciences, GlaxoSmithKline Pharmaceuticals and Janssen, and as a speaker for Abbott, AbbVie, Ascletis, Bristol‐Myers Squibb, Echosens, Gilead Sciences, Janssen, and Roche. She has also received a research grant from Gilead Sciences. Jimmy Lai has served as a speaker for Gilead Sciences and advisory committee member for Gilead Sciences and Boehringer Ingelheim. Stephen L Chan reports receiving advisory board fees from AstraZeneca, Eisai, and MSD; reports being an invited speaker for AstraZeneca, Bayer, Bristol Myers Squibb, Eisai, Ipsen, MSD, and Roche; and reports research funding (personal) from Bayer, Eisai, Ipsen, MSD, and Sirtex. Vincent Wong has served as a consultant or advisory committee member for AbbVie, AstraZeneca, Boehringer Ingelheim, Echosens, Gilead Sciences, Intercept, Inventiva, Merck, Novo Nordisk, Pfizer, Sagimet Biosciences, TARGET PharmaSolutions, and Visirna; and a speaker for Abbott, AbbVie, Echosens, Gilead Sciences, Novo Nordisk, and Unilab. He has received a research grant from Gilead Sciences, and is a cofounder of Illuminatio Medical Technology Limited. Henry Chan has served as an Independent Non‐Executive Director for Shanghai Henlius Biotech Inc.; as an advisory board member for Aligos, Arbutus, Glaxo‐Smith‐Kline, Precision Biosciences, Roche, Vaccitech, and Virion Therapeutics; and as a speaker for Echosens, Gilead, Roche, and Viatris. Terry Yip has served as an advisory committee member and a speaker for Gilead Sciences. He received a research grant from Gilead Sciences. The other authors declare no conflicts of interest.

## Supporting information


**Table S1:** List of viral serological markers retrieved.
**Table S2:** ICD‐9‐CM diagnosis and procedure codes, and ICD‐10 diagnosis codes for hepatic decompensation used internally by Hospital Authority.
**Table S3:** Drug codes of nucleos(t)ide analogues and (pegylated)‐interferon used in Hospital Authority internally.
**Table S4:** Type of other malignancies among 511 patients with other cancers at the time of receiving immune checkpoint inhibitors or tyrosine kinase inhibitors.
**Table S5:** Clinical characteristics of 25 patients at the time of hepatitis B virus reactivation as defined by the American Association for the Study of Liver Diseases (AASLD) criteria.
**Table S6:** Clinical characteristics of patients with current or past hepatitis B virus (HBV) infection and liver cancer who received immune checkpoint inhibitors (ICI) or tyrosine kinase inhibitor (TKI) and did or did not develop HBV reactivation based on the Asian Pacific Association for the Study of the Liver (APASL) criteria.
**Table S7:** Univariate and multivariable analysis with Fine‐Grey subdistribution hazard regression after multiple imputation on factors associated with the development of hepatitis B virus (HBV) reactivation in patients with current or past HBV infection and liver cancer who received immune checkpoint inhibitors (ICI) or tyrosine kinase inhibitors (TKI).
**Table S8:** Univariate and multivariable analysis with time‐dependent cause‐specific hazard regression after multiple imputation on factors associated with the development of hepatitis B virus (HBV) reactivation in patients with current or past HBV infection and liver cancer who received immune checkpoint inhibitors (ICI) or tyrosine kinase inhibitors (TKI).
**Table S9:** Clinical characteristics of patients with current or past hepatitis B virus (HBV) infection and liver cancer who received atezolizumab–bevacizumab, nivolumab–ipilimumab, or pembrolizumab and did or did not develop HBV reactivation based on the American Association for the Study of Liver Diseases criteria.
**Table S10:** Clinical characteristics of patients with current or past hepatitis B virus (HBV) infection and liver cancer who received atezolizumab–bevacizumab, nivolumab–ipilimumab, or pembrolizumab and did or did not develop HBV reactivation based on the Asian Pacific Association for the Study of the Liver criteria.
**Table S11:** Univariate and multivariable analysis with time‐dependent analysis after multiple imputation on factors associated with the development of HBV reactivation in patients with current or past infection of hepatitis B and liver cancer who received atezolizumab plus bevacizumab, nivolumab plus ipilimumab, or pembrolizumab.
**Figure S1:** Cumulative incidence of hepatitis flare in A.
**Figure S2:** Cumulative incidence of hepatic decompensation in A.

## Data Availability

The data that support the findings of this study are available on request from the corresponding author, TCFY, subject to approval from the Ethics Committee. The data are not publicly available due to ethical restrictions.
