# Supplementary material for: Comparative Risk of Hepatitis B Virus Reactivation in Patients Receiving Immune Checkpoint Inhibitors or Tyrosine Kinase Inhibitors for Liver Cancer
Source: Aliment Pharmacol Ther. 2025 Sep 5;63(3):383–95. doi: 10.1111/apt.70367 (PMC12807337; doi:10.1111/apt.70367)

**Comparative risk of hepatitis B virus reactivation in patients receiving immune checkpoint inhibitors or tyrosine kinase inhibitors for liver cancer**

**Supplementary Material**

| Table of contents | Pages |
| --- | --- |
| Supplementary Table 1. List of viral serological markers retrieved. | 3 |
| Supplementary Table 2. ICD-9-CM diagnosis and procedure codes, and ICD-10 diagnosis codes for hepatic decompensation used internally by Hospital Authority. | 4 |
| Supplementary Table 3. Drug codes of nucleos(t)ide analogues and (pegylated)-interferon used in Hospital Authority internally. | 5 |
| Supplementary Table 4. Type of other malignancies among 511 patients with other cancers at the time of receiving immune checkpoint inhibitors or tyrosine kinase inhibitors. | 6 |
| Supplementary Table 5. Clinical characteristics of 25 patients at the time of hepatitis B virus reactivation as defined by the American Association for the Study of Liver Diseases (AASLD) criteria. | 7 |
| Supplementary Table 6. Clinical characteristics of patients with current or past hepatitis B virus (HBV) infection and liver cancer who received immune checkpoint inhibitors (ICI) or tyrosine kinase inhibitor (TKI) and did or did not develop HBV reactivation based on the Asian Pacific Association for the Study of the Liver (APASL) criteria. | 8 |
| Supplementary Table 7. Univariate and multivariable analysis with Fine-Gray subdistribution hazard regression after multiple imputation on factors associated with the development of hepatitis B virus (HBV) reactivation in patients with current or past HBV infection and liver cancer who received immune checkpoint inhibitors (ICI) or tyrosine kinase inhibitors (TKI). | 9 |
| Supplementary Table 8. Univariate and multivariable analysis with time-dependent cause-specific hazard regression after multiple imputation on factors associated with the development of hepatitis B virus (HBV) reactivation in patients with current or past HBV infection and liver cancer who received immune checkpoint inhibitors (ICI) or tyrosine kinase inhibitors (TKI). | 10-11 |
| Supplementary Table 9. Clinical characteristics of patients with current or past hepatitis B virus (HBV) infection and liver cancer who received atezolizumab-bevacizumab, nivolumab-ipilimumab, or pembrolizumab and did or did not develop HBV reactivation based on the American Association for the Study of Liver Diseases criteria. | 12 |
| Supplementary Table 10. Clinical characteristics of patients with current or past hepatitis B virus (HBV) infection and liver cancer who received atezolizumab-bevacizumab, nivolumab-ipilimumab, or pembrolizumab and did or did not develop HBV reactivation based on the Asian Pacific Association for the Study of the Liver criteria. | 13 |
| Supplementary Table 11. Univariate and multivariable analysis with time-dependent analysis after multiple imputation on factors associated with the development of HBV reactivation in patients with current or past infection of hepatitis B and liver cancer who received atezolizumab plus bevacizumab, nivolumab plus ipilimumab, or pembrolizumab. | 14 |
| Supplementary Figure 1. Cumulative incidence of hepatitis flare in A. 1,596 patients with current or past HBV infection and liver cancer who received immune checkpoint inhibitors (ICI) or tyrosine kinase inhibitor (TKI), and B. 397 patients with current or past HBV infection and liver cancer who received atezolizumab/bevacizumab, nivolumab/ipilimumab, and pembrolizumab. | 15 |
| Supplementary Figure 2. Cumulative incidence of hepatic decompensation in A. 1,529 patients with current or past HBV infection and liver cancer who received immune checkpoint inhibitors (ICI) or tyrosine kinase inhibitor (TKI), and B. 369 patients with current or past HBV infection and liver cancer who received atezolizumab/bevacizumab, nivolumab/ipilimumab, and pembrolizumab. | 16 |

**Supplementary Table 1.** List of viral serological markers retrieved.

| **HBV** | **HCV** | **HDV** |
| --- | --- | --- |
| Anti-HBc | Anti-HCV | Anti-HDV |
| Anti-HBc IgM | HCV RNA (viral load), RT-PCR |  |
| Anti-HBe | HCV RNA, RT-PCR |  |
| Anti-HBs |  |  |
| Anti-HBs, Quantitative |  |  |
| HBeAg |  |  |
| HBsAg |  |  |
| HBV DNA |  |  |
| HBV DNA (viral load), RT-PCR |  |  |

Anti-HBc = antibody to hepatitis B core antigen; Anti-HBe = antibody to hepatitis B e antigen; Anti-HBs = antibody to hepatitis B surface antigen; HBeAg = hepatitis B e antigen; HBsAg = hepatitis B surface antigen; HBV = hepatitis B virus; HCV = hepatitis C virus; HDV = hepatitis D virus; IgM = immunoglobulin M; RT-PCR = Reverse transcription polymerase chain reaction.

**Supplementary Table 2.** ICD-9-CM diagnosis and procedure codes, and ICD-10 diagnosis codes for hepatic decompensation used internally by Hospital Authority.

| Diseases | ICD-9-CM codes^#^ | ICD-10 diagnosis codes | Descriptions |
| --- | --- | --- | --- |
| Ascites | 789.5 | R18, K70.31,  K70.11, K71.51 | Ascites |
| SBP | 567.2:9 | - | Spontaneous bacterial peritonitis |
| OVB* | 456.0 | I85.0 | Oesophageal varices with bleeding |
| OVB | 456.20 | I98.3 | Oesophageal varices classified elsewhere with bleeding |
| GVB* | 456.8:1 | - | Fundal varices, bleeding |
| GVB | 456.8:2 | - | Bleeding gastric varices |
| HE | 348.3 | G93.4 | Encephalopathy, unspecified |
| HE | 349.82 | G92 | Toxic encephalopathy |
| HE | 572.2 | K76.82, K72.91 | Hepatic coma |
| HRS | 572.4 | K76.7 | Hepatorenal syndrome |

^#^ Codes with the colon sign (:) referred to coding from the Hospital Authority Master Disease Code Table (HAMDCT), which extended ICD-9-CM codes by adding local terms. Reference: Fung V, Cheung NT, Szeto K, Ngai L, Lau M, Kong JH. Hospital authority clinical vocabulary table: The past, the present, and the future. Hospital Authority Clinical Vocabulary Table: the Past, the Present, and the Future/AHIMA, American Health Information Management Association. 2004 Oct 15.

* Oesophageal or gastric variceal bleeding was also defined by the ICD-9-CM procedure codes of 42.33:3, 42.33:6, 42.33:13, and 43.41:1.

^ The definition of HCC also considered the ICD-9-CM procedure codes of HCC treatment.

Abbreviations: OVB = oesophageal variceal bleeding, GVB = gastric variceal bleeding, HE = hepatic encephalopathy, HRS = hepatorenal syndrome, ICD-9-CM = International Classification of Diseases, Ninth Revision, Clinical Modification, SBP = spontaneous bacterial peritonitis.

**Supplementary Table 3.** Drug codes of nucleos(t)ide analogues and (pegylated)-interferon used in Hospital Authority internally.

| Drug code | Name | Dosage |
| --- | --- | --- |
| ADEF01 | Adefovir Dipivoxil | 10 MG |
| ENTE01 | Entecavir | 0.5 MG |
| ENTE02 | Entecavir | 1.0 MG |
| INTE04/05/18/19 | Interferon alpha-2a | 3-9MIU/0.5-1ML |
| INTE06-09/16-17 | Interferon alpha-2b | 3/5/10/15/25 MIU/1ML |
| LAMI07 | Lamivudine | 150 MG |
| LAMI08 | Lamivudine Solution | 10 MG/ML |
| LAMI09 | Lamivudine | 100 MG |
| LAMI10 | Lamivudine + Zidovudine | 150 MG/300 MG |
| PEGI01-03/05/09-12/18-21 | Peginterferon alpha-2b | 50-120 MCG/0.5-1ML |
| PEGI04/06-08/13/15-17 | Peginterferon alpha-2a | 135-180 MCG/0.5-1ML |
| PEGI14 | Peginterferon lambda-1a | 180 MCG/0.45ML |
| TELB01 | Telbivudine | 600 MG |
| TENO03/04/07 | Tenofovir Disoproxil Fumarate | 300 MG |
| TENO06/08 | Tenofovir Alafenamide | 25 MG |

**Supplementary Table 4.** Type of other malignancies among 511 patients with other cancers at the time of receiving immune checkpoint inhibitors or tyrosine kinase inhibitors.

| Type of malignancies | Number of patients (%) |
| --- | --- |
| Malignant neoplasm of lip, oral cavity, and pharynx | 5 (1.0) |
| Malignant neoplasm of digestive organs and peritoneum | 48 (9.4) |
| Malignant neoplasm of respiratory and intrathoracic organs | 26 (5.1) |
| Malignant neoplasm of bone, connective tissue and skin | 1 (0.2) |
| Malignant neoplasm of breast | 10 (2.0) |
| Kaposi’s sarcoma | 0 (0.0) |
| Malignant neoplasm of genitourinary organs | 28 (5.5) |
| Malignant neoplasm of other and unspecified sites | 437 (85.5) |
| Malignant neoplasm of lymphatic and hematopoietic tissue | 12 (2.3) |
| Personal history of malignant neoplasm | 36 (7.0) |

Some patients could have more than one type of malignancy.

**Supplementary Table 5.** Clinical characteristics of 25 patients at the time of hepatitis B virus reactivation as defined by the American Association for the Study of Liver Diseases (AASLD) criteria.

| Patient | Systemic therapies | HBV antiviral prophylaxis | Age  (years) | Sex | Current/past HBV | HBsAg status | HBV DNA level (IU/mL) | ALT (U/L) | Albumin (g/L) | Total bilirubin (µmol/L) | Occurrence of hepatic decompensation after HBV reactivation |
| --- | --- | --- | --- | --- | --- | --- | --- | --- | --- | --- | --- |
| 1 | Lenvatinib | Yes | 70 | M | Past HBV | Negative | Detected, <10 IU/mL | 827 | 26 | 506 | No |
| 2 | Lenvatinib*  🡪pembrolizumab | Yes | 56 | M | Current HBV | Positive | 78200 | 134 | 21 | 17 | No |
| 3 | Lenvatinib | Yes | 66 | M | Past HBV | Negative | 88 | 65 | 16 | 198 | No |
| 4 | Atezolizumab/bevacizumab*  🡪nivolumab/ipilimumab  🡪pembrolizumab  🡪cabozantinib | Yes | 53 | M | Past HBV | Negative | 28 | 101 | 39 | 15 | No |
| 5 | Lenvatinib | Yes | 68 | M | Current HBV | Positive | 15400 | 190 | 16 | 142 | No |
| 6 | Lenvatinib | Yes | 71 | M | Current HBV | Positive | 1200 | 65 | 34 | 324 | No |
| 7 | Lenvatinib | No | 77 | M | Past HBV | Negative | 39 | 34 | 27 | 11 | No |
| 8 | Nivolumab/ipilimumab | No | 67 | M | Past HBV | Negative | Detected, <10 IU/mL | 289 | 13 | 81 | No |
| 9 | Lenvatinib | Yes | 46 | M | Past HBV | Negative | 19 | 251 | - | - | No |
| 10 | Sorafenib | No | 69 | F | Past HBV | Negative | Detected, <10 IU/mL | 388 | 37 | 12 | No |
| 11 | Lenvatinib | Yes | 64 | M | Past HBV | Negative | 512 | 215 | 15 | 531 | No |
| 12 | Lenvatinib | Yes | 67 | F | Past HBV | Negative | 24 | 91 | 21 | 81 | No |
| 13 | Lenvatinib | Yes | 50 | M | Current HBV | Positive | 18400 | 124 | 15 | 132 | Ascites |
| 14 | Lenvatinib | Yes | 58 | M | Past HBV | Negative | 80 | 142 | 27 | 62 | No |
| 15 | Lenvatinib | Yes | 70 | M | Past HBV | Positive | - | 285 | 16 | 162 | No |
| 16 | Atezolizumab/bevacizumab | Yes | 66 | M | Past HBV | Negative | 474 | 40 | 29 | 22 | No |
| 17 | Sorafenib | Yes | 70 | M | Past HBV | Positive | - | 28 | 23 | 11 | No |
| 18 | Lenvatinib | Yes | 53 | M | Past HBV | Negative | 577 | 105 | 20 | 72 | No |
| 19 | Sorafenib | Yes | 61 | M | Past HBV | Positive | - | 87 | - | - | No |
| 20 | Lenvatinib | Yes | 68 | M | Current HBV | Positive | 2190000 | 248 | 28 | 10 | No |
| 21 | Lenvatinib | Yes | 57 | M | Current HBV | Positive | 6420 | 397 | - | - | No |
| 22 | Lenvatinib | Yes | 63 | M | Past HBV | Negative | 7740 | 44 | 32 | 192 | No |
| 23 | Lenvatinib  🡪pembrolizumab* | Yes | 61 | M | Current HBV | Positive | 31300 | 5706 | 23 | 362 | Ascites |
| 24 | Lenvatinib*  🡪sorafenib  🡪pembrolizumab | No | 51 | M | Past HBV | Negative | 52 | 280 | 35 | 23 | No |
| 25 | Sorafenib | Yes | 61 | M | Current HBV | Positive | 40200 | 392 | 12 | 13 | No |

* HBV reactivation occurred during this systemic therapy.

**Supplementary Table 6.** Clinical characteristics of patients with current or past hepatitis B virus (HBV) infection and liver cancer who received immune checkpoint inhibitors (ICI) or tyrosine kinase inhibitor (TKI) and did or did not develop HBV reactivation based on the Asian Pacific Association for the Study of the Liver (APASL) criteria.

| Clinical characteristics | Without Reactivation  N=1,561 | With Reactivation  N=35 | p value |
| --- | --- | --- | --- |
| Age (years) | 63.3 ± 10.8 | 62.6 ± 8.7 | 0.682 |
| Men (n, %) | 1,337 (85.7) | 29 (82.9) | 0.642 |
| Presence of other cancers (n, %) | 500 (32.0) | 11 (31.4) | 0.940 |
| HBV infection (n, %) |  |  | <0.001 |
| - Current HBV | 1,373 (88.0) | 18 (51.4) |  |
| - Past HBV | 188 (12.0) | 17 (48.6) |  |
| HBV antiviral prophylaxis (n, %) | 1,456 (93.3) | 31 (88.6) | 0.295 |
| HCV coinfection, (%) | 61 (5.3) | 0 (0) | 0.394 |
| Alanine aminotransferase (U/L) | 38 (25-64) | 41 (23-59) | 0.905 |
| Missing (%) | 0.1 | 0 |  |
| HBV DNA level (log_10_IU/mL) | 3.8 ± 4.9 | 1.7 ± 2.5 | 0.020 |
| Missing (%) | 24 | 20 |  |
| HBV DNA level of patients with current HBV infection (log_10_IU/mL) | 4.2 ± 4.9 | 0.5 ± 1.3 | <0.001 |
| Missing (%) | 22.1 | 11.1 |  |
| Use of liver cancer TKI before baseline (n, %) | 1,344 (86.1) | 30 (85.7) | >0.999 |
| Start of liver cancer TKI in follow-up (n, %) | 69 (4.4) | 1 (2.9) | >0.999 |
| Use of ICI at baseline, n (%) | 217 (13.9) | 5 (14.3) | >0.999 |
| Switch to ICI in follow-up, n (%) | 173 (11.1) | 2 (5.7) | 0.420 |
| Use of other target therapies before baseline (n, %) | 16 (1.0) | 1 (2.9) | 0.315 |
| Start of other target therapies in follow-up (n, %) | 141 (9.0) | 1 (2.9) | 0.361 |
| Use of other chemotherapy before baseline (n, %) | 541 (34.7) | 14 (40.0) | 0.512 |
| Start of other chemotherapy in follow-up (n, %) | 72 (4.6) | 1 (2.9) | >0.999 |
| Liver resection before baseline (n, %) | 150 (9.6) | 5 (14.3) | 0.379 |
| LAT before baseline (n, %) | 82 (5.3) | 2 (5.7) | 0.706 |
| TACE before baseline (n, %) | 527 (33.8) | 14 (40.0) | 0.441 |
| Use of steroid/IS before baseline (n, %) | 385 (24.7) | 12 (34.3) | 0.193 |
| Start of steroid/IS in follow-up (n, %) | 310 (19.9) | 4 (11.4) | 0.215 |
| Follow-up duration (months) | 10.8 (3.7-12.0) | 5.3 (2.3-8.5) | <0.001 |

HBV=hepatitis B virus; HCV=hepatitis C virus; IS=immunosuppressants; LAT=local ablation therapy; TACE=transarterial chemoembolization; TKI=tyrosine kinase inhibitors

**Supplementary Table 7.** Univariate and multivariable analysis with Fine-Gray subdistribution hazard regression after multiple imputation on factors associated with the development of hepatitis B virus (HBV) reactivation in patients with current or past HBV infection and liver cancer who received immune checkpoint inhibitors (ICI) or tyrosine kinase inhibitors (TKI).

| HBV reactivation using AASLD criteria (reactivation rate=25/1,596 [1.6%]) | | | | |
| --- | --- | --- | --- | --- |
| Parameters | **Univariate analysis** | | **Multivariable analysis** | |
|  | **sHR (95% CI)** | **p value** | **aSHR (95% CI)** | **p value** |
| ICI vs TKI | 0.90 (0.27-3.01) | 0.870 | 1.03 (0.30-3.54) | 0.962 |
| Past vs current HBV infection | 15.59 (6.75-36.00) | <0.001 |  |  |
| HBV antiviral prophylaxis | 0.38 (0.13-1.11) | 0.076 | 0.38 (0.13-1.14) | 0.085 |
| Age (years) | 0.99 (0.97-1.02) | 0.497 |  |  |
| Men | 1.94 (0.46-8.19) | 0.370 |  |  |
| Presence of other cancers | 0.66 (0.26-1.64) | 0.370 |  |  |
| Alanine aminotransferase (U/L) | 1.00 (1.00-1.01) | 0.150 |  |  |
| HBV DNA (log_10_IU/mL) | 0.99 (0.91-1.07) | 0.813 |  |  |
| Other chemotherapy before baseline | 0.60 (0.24-1.51) | 0.282 |  |  |
| TACE before baseline | 0.63 (0.25-1.58) | 0.329 |  |  |
| Steroid/immunosuppressant use before baseline | 1.14 (0.48-2.73) | 0.761 |  |  |
| HBV reactivation using APASL criteria (reactivation rate=35/1,596 [2.2%]) | | | | |
| Parameters | **Univariate analysis** | | **Multivariable analysis** | |
|  | **sHR (95% CI)** | **p value** | **aSHR (95% CI)** | **p value** |
| ICI vs TKI | 1.12 (0.44-2.87) | 0.815 | 0.86 (0.33-2.25) | 0.762 |
| Past vs current HBV infection | 6.98 (3.62-13.46) | <0.001 |  |  |
| HBV antiviral prophylaxis | 0.56 (0.20-1.59) | 0.277 | 0.55 (0.19-1.60) | 0.276 |
| Age (years) | 1.00 (0.97-1.02) | 0.705 |  |  |
| Male gender | 0.81 (0.34-1.94) | 0.634 |  |  |
| Presence of other cancers | 0.96 (0.47-1.95) | 0.902 |  |  |
| Alanine aminotransferase (U/L) | 1.00 (0.99-1.00) | 0.639 |  |  |
| HBV DNA (log_10_IU/mL) | 0.91 (0.82-1.00) | 0.060 |  |  |
| Other chemotherapy before baseline | 1.29 (0.66-2.53) | 0.462 |  |  |
| TACE before baseline | 1.35 (0.69-2.65) | 0.382 |  |  |
| Steroid/immunosuppressant use before baseline | 1.54 (0.77-3.07) | 0.225 |  |  |

HBV antiviral prophylaxis was forced into the model for multivariable adjustment. Past vs current HBV infection was not put into the multivariable analysis due to a significant correlation with HBV antiviral prophylaxis.

aSHR=adjusted subdistribution hazard ratio; AASLD=American Association for the Study of Liver Diseases; APASL= Asian Pacific Association for the Study of the Liver; HBV=hepatitis B virus; ICI=immune checkpoint inhibitors; TACE=transarterial chemoembolization; TKI=tyrosine kinase inhibitors

**Supplementary Table 8.** Univariate and multivariable analysis with time-dependent cause-specific hazard regression after multiple imputation on factors associated with the development of hepatitis B virus (HBV) reactivation in patients with current or past HBV infection and liver cancer who received immune checkpoint inhibitors (ICI) or tyrosine kinase inhibitors (TKI).

|  | **HBV reactivation by AASLD criteria** | | | | **Competing mortality** | | | |
| --- | --- | --- | --- | --- | --- | --- | --- | --- |
| **Parameters** | **Univariate analysis** | | **Multivariable analysis** | | **Univariate analysis** | | **Multivariable analysis** | |
|  | **CSHR (95% CI)** | **p value** | **aCSHR (95% CI)** | **p value** | **CSHR (95% CI)** | **p value** | **aCSHR (95% CI)** | **p value** |
| Change of systemic therapy class |  |  |  |  |  |  |  |  |
| TKI only | Reference |  |  |  | Reference |  |  |  |
| ICI only | 1.27 (0.35-4.61) | 0.705 | 1.36 (0.37-4.97) | 0.630 | 1.03 (0.79-1.33) | 0.833 | 1.08 (0.83-1.40) | 0.579 |
| TKI to ICI or ICI to TKI | 1.22 (0.25-5.80) | 0.797 | 1.33 (0.28-6.44) | 0.707 | 1.85 (1.43-2.39) | <0.001 | 1.86 (1.43-2.42) | <0.001 |
| Past vs current HBV infection | 13.77 (5.66-33.48) | <0.001 |  |  | 0.57 (0.43-0.75) | <0.001 |  |  |
| HBV antiviral prophylaxis | 0.40 (0.13-1.25) | 0.112 | 0.39 (0.12-1.23) | 0.102 | 1.35 (0.96-1.89) | 0.088 | 0.98 (0.69-1.39) | 0.903 |
| Age (years) | 0.99 (0.95-1.03) | 0.493 |  |  | 0.99 (0.98-0.99) | <0.001 |  |  |
| Men | 1.95 (0.42-8.98) | 0.375 |  |  | 1.03 (0.83-1.29) | 0.774 |  |  |
| Presence of other cancers | 0.74 (0.28-1.96) | 0.532 |  |  | 1.49 (1.27-1.74) | <0.001 | 1.56 (1.33-1.83) | <0.001 |
| Alanine aminotransferase (U/L) | 1.00 (1.00-1.01) | 0.150 |  |  | 1.004 (1.003-1.005) | <0.001 | 1.003 (1.002-1.004) | <0.001 |
| HBV DNA (log_10_IU/mL) | 1.00 (0.91-1.11) | 0.923 |  |  | 1.07 (1.05-1.09) | <0.001 | 1.06 (1.04-1.08) | <0.001 |
| Other chemotherapy before baseline | 0.58 (0.22-1.52) | 0.253 |  |  | 0.91 (0.77-1.07) | 0.245 |  |  |
| TACE before baseline | 0.61 (0.23-1.60) | 0.299 |  |  | 0.94 (0.80-1.11) | 0.464 |  |  |
| Steroid/other IS use before baseline | 1.10 (0.44-2.78) | 0.828 |  |  | 0.91 (0.76-1.09) | 0.322 |  |  |
|  | **HBV reactivation by APASL criteria** | | | | **Competing mortality** | | | |
| **Parameters** | **Univariate analysis** | | **Multivariable analysis** | | **Univariate analysis** | | **Multivariable analysis** | |
|  | **CSHR (95% CI)** | **p value** | **aCSHR (95% CI)** | **p value** | **CSHR (95% CI)** | **p value** | **aCSHR (95% CI)** | **p value** |
| Change of systemic therapy class |  |  |  |  |  |  |  |  |
| TKI only | Reference |  |  |  | Reference |  |  |  |
| ICI only | 1.24 (0.42-3.69) | 0.694 | 1.28 (0.43-3.82) | 0.652 | 1.03 (0.79-1.34) | 0.820 | 1.11 (0.85-1.44) | 0.455 |
| TKI to ICI or ICI to TKI | 1.22 (0.35-4.26) | 0.752 | 1.27 (0.36-4.48) | 0.700 | 1.88 (1.45-2.43) | <0.001 | 1.89 (1.45-2.45) | <0.001 |
| Past vs current HBV infection | 6.08 (3.05-12.10) | <0.001 |  |  | 0.57 (0.43-0.75) | <0.001 |  |  |
| HBV antiviral prophylaxis | 0.60 (0.20-1.78) | 0.346 | 0.58 (0.20-1.74) | 0.322 | 1.35 (0.96-1.90) | 0.086 | 0.93 (0.66-1.33) | 0.709 |
| Age (years) | 0.99 (0.96-1.02) | 0.564 |  |  | 0.99 (0.98-0.99) | <0.001 |  |  |
| Men | 0.82 (0.33-2.04) | 0.658 |  |  | 1.06 (0.84-1.32) | 0.632 |  |  |
| Presence of other cancers | 1.09 (0.52-2.30) | 0.809 |  |  | 1.49 (1.27-1.74) | <0.001 | 1.56 (1.33-1.83) | <0.001 |
| Alanine aminotransferase (U/L) | 1.00 (0.99-1.01) | 0.863 |  |  | 1.00 (1.00-1.00) | <0.001 | 1.003 (1.002-1.004) | <0.001 |
| HBV DNA (log_10_IU/mL) | 0.92 (0.82-1.03) | 0.141 |  |  | 1.08 (1.06-1.10) | <0.001 | 1.08 (1.06-1.10) | <0.001 |
| Other chemotherapy before baseline | 1.22 (0.60-2.47) | 0.567 |  |  | 0.89 (0.76-1.05) | 0.174 |  |  |
| TACE before baseline | 1.29 (0.64-2.61) | 0.466 |  |  | 0.92 (0.78-1.09) | 0.352 |  |  |
| Steroid/other IS use before baseline | 1.47 (0.71-3.04) | 0.285 |  |  | 0.91 (0.76-1.09) | 0.293 |  |  |

HBV antiviral prophylaxis was forced into the model for multivariable adjustment. Past vs current HBV infection was not put into the multivariable analysis due to a significant correlation with HBV antiviral prophylaxis.

AASLD=American Association for the Study of Liver Diseases; APASL= Asian Pacific Association for the Study of the Liver; aCSHR= adjusted cause-specific hazards ratio; HBV=hepatitis B virus; ICI=immune checkpoint inhibitors; IS=immunosuppressants; TACE=transarterial chemoembolization; TKI=tyrosine kinase inhibitors

**Supplementary Table 9.** Clinical characteristics of patients with current or past hepatitis B virus (HBV) infection and liver cancer who received atezolizumab-bevacizumab, nivolumab-ipilimumab, or pembrolizumab and did or did not develop HBV reactivation based on the American Association for the Study of Liver Diseases criteria.

| **Clinical characteristics** | **Without Reactivation**  **N=391** | **With Reactivation**  **N=6** | **p value** |
| --- | --- | --- | --- |
| Age (years) | 62.0 ± 11.2 | 66.7 ± 11.2 | 0.306 |
| Men, n (%) | 333 (85.2) | 6 (100) | 0.599 |
| Presence of other cancers, n (%) | 128 (32.7) | 2 (33.3) | >0.999 |
| HBV infection, n (%) |  |  | 0.001 |
| - Current HBV | 353 (90.3) | 2 (33.3) |  |
| - Past HBV | 38 (9.7) | 4 (66.7) |  |
| HBV antiviral prophylaxis, n (%) | 383 (98.0) | 4 (66.7) | 0.008 |
| HCV coinfection, n (%) | 10 (3.5) | 0 (0) | >0.999 |
| ALT (U/L) | 41 (26-69) | 66 (35-92) | 0.380 |
| Missing (%) | 0.3 | 0 |  |
| HBV DNA level of patients with current HBV infection (log_10_IU/mL) | 3.6 ± 4.3 | 3.5 ± 1.2 | 0.984 |
| Missing (%) | 24.9 | 0.0 |  |
| Switch of ICI in follow-up, n (%) | 140 (35.8) | 1 (16.7) | 0.429 |
| Use of liver cancer target therapy before ICI, n (%) | 161 (41.2) | 3 (50.0) | 0.694 |
| Start of liver cancer target therapy in follow-up, n (%) | 75 (19.2) | 0 (0) | 0.599 |
| Use of other target therapies before ICI, n (%) | 12 (3.1) | 0 (0) | >0.999 |
| Start of other target therapies in follow-up, n (%) | 53 (13.6) | 1 (16.7) | 0.587 |
| Use of other chemotherapy before ICI, n (%) | 109 (27.9) | 4 (66.7) | 0.058 |
| Start of other chemotherapy in follow-up, n (%) | 22 (5.6) | 0 (0) | >0.999 |
| Liver resection before ICI, n (%) | 34 (8.7) | 0 (0) | >0.999 |
| LAT before ICI, n (%) | 15 (3.8) | 0 (0) | >0.999 |
| TACE before ICI, n (%) | 98 (25.1) | 4 (66.7) | 0.040 |
| Use of steroid/IS before ICI, n (%) | 91 (23.3) | 3 (50.0) | 0.148 |
| Start of steroid/IS in follow-up, n (%) | 90 (23.0) | 0 (0) | 0.344 |
| Follow-up duration (months) | 7.0 (2.3-12.0) | 2.3 (2.1-6.2) | 0.173 |

ALT=alanine transaminase; HBV=hepatitis B virus; HCV=hepatitis C virus; ICI=immune checkpoint inhibitors; IS=immunosuppressants; LAT=local ablation therapy; TACE=transarterial chemoembolization; TKI=tyrosine kinase inhibitors

**Supplementary Table 10.** Clinical characteristics of patients with current or past hepatitis B virus (HBV) infection and hepatocellular carcinoma who received atezolizumab-bevacizumab, nivolumab-ipilimumab, or pembrolizumab and did or did not develop HBV reactivation based on the Asian Pacific Association for the Study of the Liver criteria.

| **Clinical characteristics** | **Without Reactivation**  **N=387** | **With Reactivation N=10** | **p value** |
| --- | --- | --- | --- |
| Age (years) | 62.0 ± 11.3 | 63.9 ± 9.9 | 0.596 |
| Men, n (%) | 329 (85.0) | 10 (100) | 0.370 |
| Presence of other cancers, n (%) | 128 (33.1) | 2 (20.0) | 0.509 |
| HBV infection, n (%) |  |  | 0.014 |
| - Current HBV | 349 (90.2) | 6 (60.0) |  |
| - Past HBV | 38 (9.8) | 4 (40.0) |  |
| HBV antiviral prophylaxis, n (%) | 379 (97.9) | 8 (80.0) | 0.023 |
| HCV coinfection, n (%) | 10 (3.5) | 0 (0) | >0.999 |
| ALT (U/L) | 41 (26-69) | 37 (25-81) | 0.918 |
| Missing (%) | 0.3 | 0 |  |
| HBV DNA level of patients with current HBV infection (log_10_IU/mL) | 3.6 ± 4.4 | 1.2 ± 1.9 | 0.022 |
| Missing (%) | 25.2 | 0.0 |  |
| Switch of ICI in follow-up, n (%) | 138 (35.7) | 2 (20.0) | 0.505 |
| Use of liver cancer target therapy before ICI, n (%) | 159 (41.1) | 5 (50.0) | 0.747 |
| Start of liver cancer target therapy in follow-up, n (%) | 73 (18.9) | 1 (10.0) | 0.696 |
| Use of other target therapies before ICI, n (%) | 12 (3.1) | 0 (0) | >0.999 |
| Start of other target therapies in follow-up, n (%) | 53 (13.7) | 1 (10.0) | >0.999 |
| Use of other chemotherapy before ICI, n (%) | 108 (27.9) | 5 (50.0) | 0.156 |
| Start of other chemotherapy in follow-up, n (%) | 22 (5.7) | 0 (0) | >0.999 |
| Liver resection before ICI, n (%) | 34 (8.8) | 0 (0) | >0.999 |
| LAT before ICI, n (%) | 15 (3.9) | 0 (0) | >0.999 |
| TACE before ICI, n (%) | 97 (25.1) | 5 (50.0) | 0.133 |
| Use of steroid/IS before ICI, n (%) | 91 (23.5) | 3 (30.0) | 0.706 |
| Start of steroid/IS in follow-up, n (%) | 89 (23.0) | 1 (10.0) | 0.467 |
| Follow-up duration (months) | 7.0 (2.3-12.0) | 2.3 (1.8-5.8) | 0.044 |

ALT=alanine transaminase; HBV=hepatitis B virus; HCV=hepatitis C virus; ICI=immune checkpoint inhibitors; IS=immunosuppressants; LAT=local ablation therapy; TACE=transarterial chemoembolization; TKI=tyrosine kinase inhibitors

**Supplementary Table 11.** Univariate and multivariable analysis with time-dependent analysis after multiple imputation on factors associated with the development of HBV reactivation in patients with current or past infection of hepatitis B and liver cancer who received atezolizumab plus bevacizumab, nivolumab plus ipilimumab, or pembrolizumab.

|  | **HBV reactivation** | | | | **Competing mortality** | | | |
| --- | --- | --- | --- | --- | --- | --- | --- | --- |
|  | **HBV reactivation using AASLD 2018 guideline (reactivation rate=6/397 [1.5%])** | | | | | | | |
| **Parameters** | **Univariate analysis** | | **Multivariable analysis** | | **Univariate analysis** | | **Multivariable analysis** | |
|  | **CSHR (95% CI)** | **p value** | **aCSHR (95% CI)** | **p value** | **CSHR (95% CI)** | **p value** | **aCSHR (95% CI)** | **p value** |
| Change of ICI therapies* | - | - | - | - | 1.94 (0.94-4.01) | 0.074 | 2.16 (1.04 - 4.48) | 0.039 |
| HBV infection |  |  |  |  |  |  |  |  |
| - Chronic HBV | Reference |  |  |  | Reference |  |  |  |
| - Resolved HBV | 16.90 (3.09-92.37) | 0.034 |  |  | 0.59 (0.32-1.09) | 0.093 |  |  |
| HBV antiviral prophylaxis | 0.05 (0.00-0.62) | 0.030 | 0.05 (0.00-0.62) | 0.030 | 1.09 (0.40-2.95) | 0.871 | 0.90 (0.32 - 2.47) | 0.831 |
| Age | 1.04 (0.93-1.16) | 0.411 |  |  | 0.99 (0.97-1.00) | 0.027 |  |  |
| Men^ | - | - |  |  | 1.06 (0.68-1.65) | 0.799 |  |  |
| Presence of other cancers | 1.10 (0.09-12.99) | 0.921 |  |  | 1.48 (1.09-2.02) | 0.013 | 1.75 (1.27 - 2.41) | <0.001 |
| ALT at ICI (U/L) | 1.01 (0.99-1.02) | 0.394 |  |  | 1.01 (1.00-1.01) | <0.001 | 1.01 (1.00 - 1.01) | <0.001 |
| HBV DNA at ICI (log_10_IU/mL) | 1.02 (0.74-1.39) | 0.892 |  |  | 1.06 (1.02-1.10) | 0.002 | 1.05 (1.01 - 1.09) | 0.008 |
| Other chemotherapy | 5.06 (0.43-59.79) | 0.139 |  |  | 1.06 (0.76-1.48) | 0.736 |  |  |
| TACE before ICI | 6.94 (0.58-82.44) | 0.094 |  |  | 1.34 (0.96-1.88) | 0.085 | 1.50 (1.06 - 2.12) | 0.021 |
| Steroid/immunosuppressant use | 2.98 (0.29-30.58) | 0.257 |  |  | 1.03 (0.73-1.46) | 0.853 |  |  |
|  | **HBV reactivation using APASL 2016 guideline (reactivation rate=10/397 [2.5%])** | | | | | | | |
| **Parameters** | **Univariate analysis** | | **Multivariable analysis** | | **Univariate analysis** | | **Multivariable analysis** | |
|  | **CSHR (95% CI)** | ***P* value** | **aCSHR (95% CI)** | ***P* value** | **CSHR (95% CI)** | ***P* value** | **aCSHR (95% CI)** | ***P* value** |
| Change of ICI therapies* | - | - | - | - | 1.95 (0.94-4.03) | 0.073 | 2.12 (1.02-4.43) | 0.045 |
| HBV infection |  |  |  |  |  |  |  |  |
| - Chronic HBV | Reference |  |  |  | Reference |  |  |  |
| - Resolved HBV | 5.55 (1.23-25.04) | 0.031 |  |  | 0.59 (0.32-1.09) | 0.094 |  |  |
| HBV antiviral prophylaxis | 0.11 (0.02-0.68) | 0.024 | 0.11 (0.02-0.68) | 0.024 | 1.08 (0.40-2.94) | 0.874 | 0.85 (0.31-2.34) | 0.748 |
| Age | 1.01 (0.95-1.08) | 0.708 |  |  | 0.99 (0.97-1.00) | 0.03 |  |  |
| Men^ | - | - |  |  | 1.06 (0.68-1.65) | 0.803 |  |  |
| Presence of other cancers | 0.54 (0.09-3.43) | 0.463 |  |  | 1.49 (1.09-2.03) | 0.013 | 1.75 (1.27-2.41) | <0.001 |
| ALT at ICI (U/L) | 1.00 (0.99-1.02) | 0.798 |  |  | 1.01 (1.00-1.01) | <0.001 | 1.01 (1.00-1.01) | <0.001 |
| HBV DNA at ICI (log_10_IU/mL) | 0.92 (0.72-1.17) | 0.443 |  |  | 1.07 (1.04-1.11) | <0.001 | 1.07 (1.03-1.11) | <0.001 |
| Other chemotherapy | 2.53 (0.58-11.07) | 0.183 |  |  | 1.07 (0.76-1.49) | 0.707 |  |  |
| TACE before ICI | 3.43 (0.78-15.06) | 0.090 |  |  | 1.35 (0.97-1.89) | 0.078 | 1.54 (1.09-2.18) | 0.014 |
| Steroid/immunosuppressant use | 1.29 (0.26-6.43) | 0.726 |  |  | 1.04 (0.73-1.47) | 0.846 |  |  |

* Among the patients who developed HBV reactivation in 12 months, no one switched ICI therapies.

^ Among the patients who developed HBV reactivation in 12 months, all were men.

AASLD=American Association for the Study of Liver Diseases; APASL= Asian Pacific Association for the Study of the Liver; aCSHR= adjusted cause-specific hazards ratio; HBV=hepatitis B virus; ICI=immune checkpoint inhibitors; IS=immunosuppressants; TACE=transarterial chemoembolization; TKI=tyrosine kinase inhibitors

**Supplementary Figure 1.** Cumulative incidence of hepatitis flare in A. 1,596 patients with current or past HBV infection and liver cancer who received immune checkpoint inhibitors (ICI) or tyrosine kinase inhibitor (TKI), and B. 397 patients with current or past HBV infection and liver cancer who received atezolizumab/bevacizumab, nivolumab/ipilimumab, and pembrolizumab.

A.


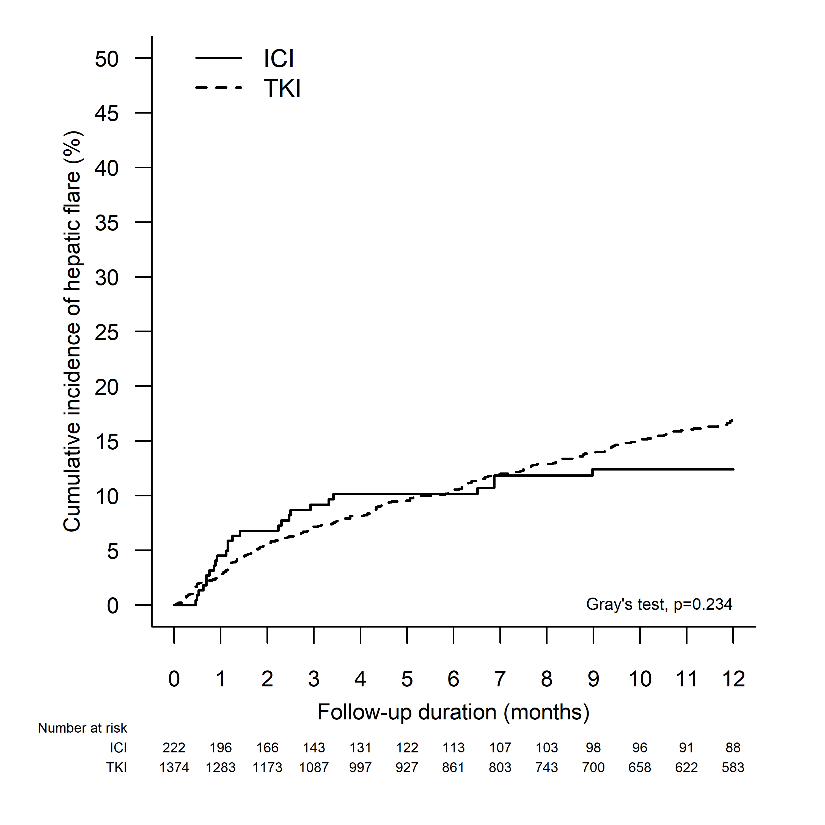


B.


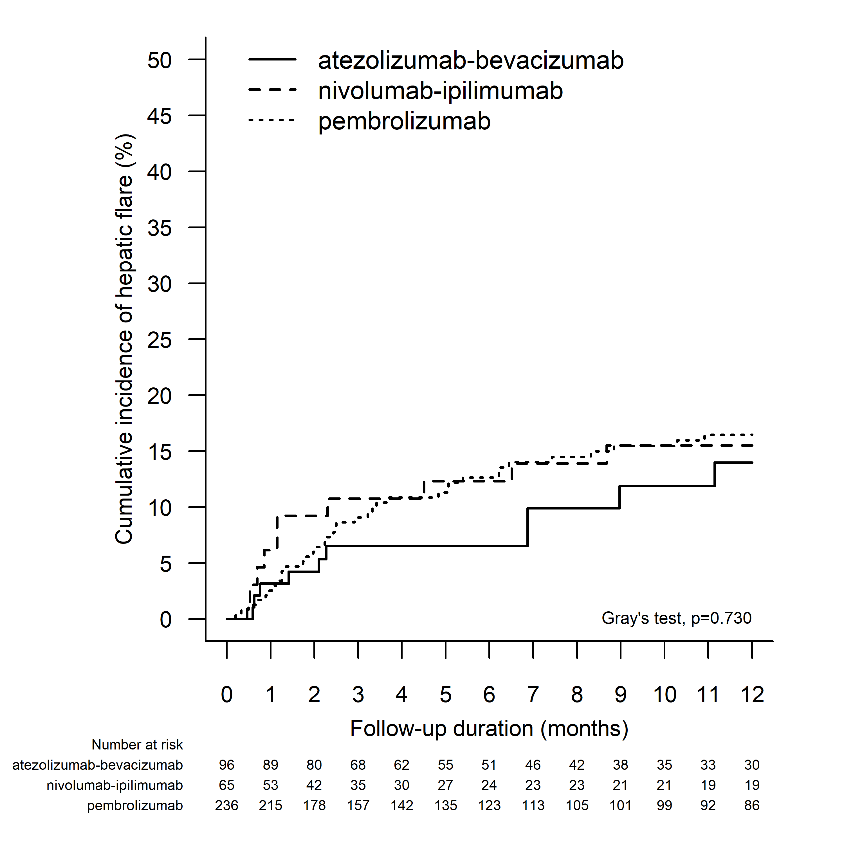


**Supplementary Figure 2.** Cumulative incidence of hepatic decompensation in A. 1,529 patients with current or past HBV infection and liver cancer who received immune checkpoint inhibitors (ICI) or tyrosine kinase inhibitor (TKI), and B. 369 patients with current or past HBV infection and liver cancer who received atezolizumab/bevacizumab, nivolumab/ipilimumab, and pembrolizumab.

A.


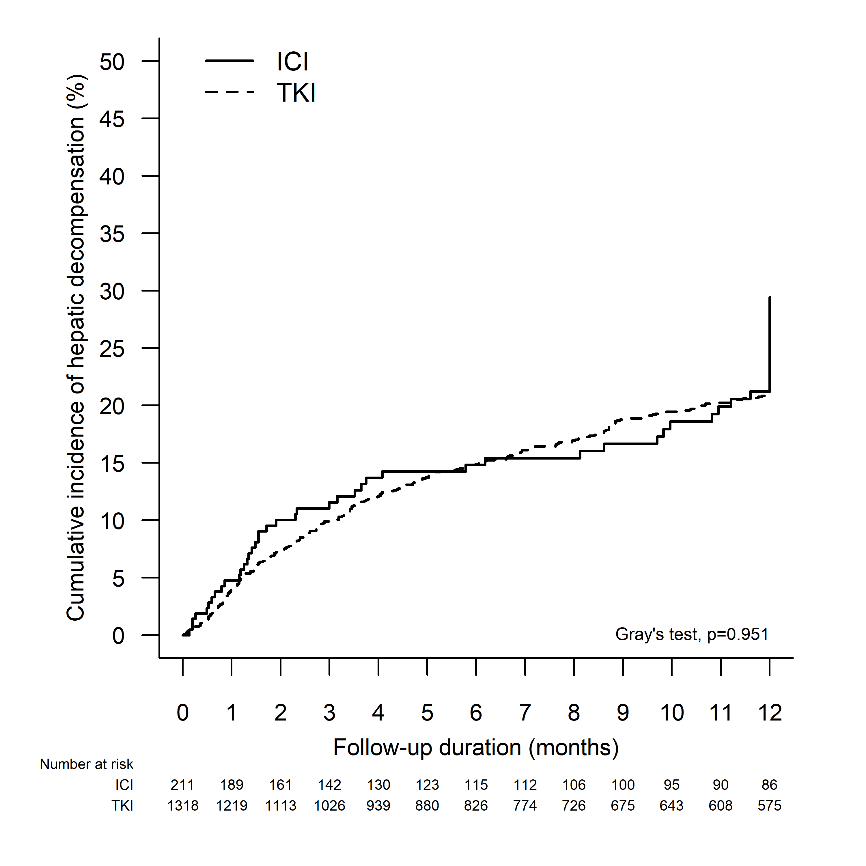


B.


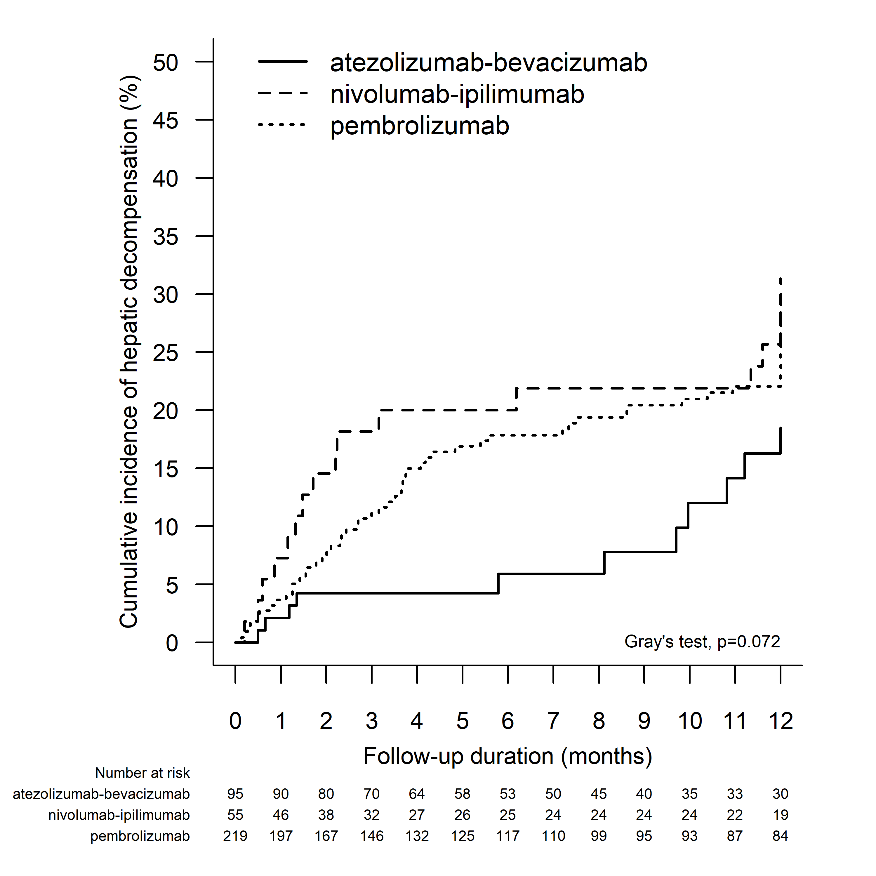

Supplement: Supplementary file 1 — Table S1: List of viral serological markers retrieved. Table S2: ICD‐9‐CM diagnosis and procedure codes, and ICD‐10 diagnosis codes for hepatic decompensation used internally by Hospital Authority. Table S3: Drug codes of nucleos(t)ide analogues and (pegylated)‐interferon used in Hospital Authority internally. Table S4: Type of other malignancies among 511 patients with other cancers at the time of receiving immune checkpoint inhibitors or tyrosine kinase inhibitors. Table S5: Clinical characteristics of 25 patients at the time of hepatitis B virus reactivation as defined by the American Association for the Study of Liver Diseases (AASLD) criteria. Table S6: Clinical characteristics of patients with current or past hepatitis B virus (HBV) infection and liver cancer who received immune checkpoint inhibitors (ICI) or tyrosine kinase inhibitor (TKI) and did or did not develop HBV reactivation based on the Asian Pacific Association for the Study of the Liver (APASL) criteria. Table S7: Univariate and multivariable analysis with Fine‐Grey subdistribution hazard regression after multiple imputation on factors associated with the development of hepatitis B virus (HBV) reactivation in patients with current or past HBV infection and liver cancer who received immune checkpoint inhibitors (ICI) or tyrosine kinase inhibitors (TKI). Table S8: Univariate and multivariable analysis with time‐dependent cause‐specific hazard regression after multiple imputation on factors associated with the development of hepatitis B virus (HBV) reactivation in patients with current or past HBV infection and liver cancer who received immune checkpoint inhibitors (ICI) or tyrosine kinase inhibitors (TKI). Table S9: Clinical characteristics of patients with current or past hepatitis B virus (HBV) infection and liver cancer who received atezolizumab–bevacizumab, nivolumab–ipilimumab, or pembrolizumab and did or did not develop HBV reactivation based on the American Association for the Study of Liver D [file APT-63-383-s001.docx]
